# Species, cell proliferation state and oxygen partial pressure are major factors in the in vitro toxicity of repurposed potential ADPKD drugs in human and murine renal proximal tubule cells

**DOI:** 10.1007/s00204-026-04472-2

**Published:** 2026-06-27

**Authors:** Paraskevi Sotiropoulou, Andrea Gerdemann, Stefan Jovanovic, Hans-Ulrich Humpf, Alexander Bürkle, Daniel R. Dietrich

**Affiliations:** 1 Human and Environmental Toxicology, University of Konstanz, Konstanz, Germany; 2 Institute of Food Chemistry, University of Münster, Münster, Germany; 3 Molecular Toxicology, University of Konstanz, Konstanz, Germany

**Keywords:** Renal proximal tubular cell toxicity, Drug repurposing, Polycystic kidney disease, Oxidative stress, Physiological oxygen, Cell proliferation

## Abstract

Unexpected nephrotoxicity during clinical stages of drug development, due to undetected toxicity in in vitro and in vivo settings, can lead to drug attrition and jeopardize drug development. The latter highlights the importance of in vitro renal toxicity testing that can safely be translated to humans. Two factors commonly overlooked in the latter are the continuous cell proliferation that occurs in regeneration processes after renal insult and the physiological O_2_ tension in the kidney. Autosomal Dominant Polycystic Kidney Disease is a genetic disorder characterized by the development of renal cysts and cellular changes, i.e. abnormal proliferation in conjunction with the Warburg effect. There is only one drug approved for the disease, tolvaptan, which, however, presents with liver toxicity and polyuria. In a quest for potential alternatives to tolvaptan, we compared the nephrotoxic and metabolic effects of four repurposed drugs, salicylic acid (up to 10 mM), birinapant (up to 100 µM), bardoxolone methyl (up to 1000 nM), and rapamycin (up to 160 nM) to tolvaptan (up to 100 µM) on human differentiated and proliferating (RPTEC/TERT1) and mouse proliferating (mProx24) renal proximal tubular epithelial cells at atmospheric (21%) and physiological (10%) O_2_ tensions. The focus was on improved interpretation of in vitro test results for the detection of potential adverse effects. We found that cell proliferation and O_2_ tension are major factors that determine the susceptibility of cells to compounds, while the drug effects on the metabolism and mitochondrial function provided further insight into the mechanisms underlying the observed in vitro toxicity.

**Supplementary Information:**

The online version contains supplementary material available at https://doi.org/10.1007/s00204-026-04472-2.

## Introduction

The renal proximal tubule is considered the gold standard for in vitro nephrotoxicity testing, as it is lined by simple cuboidal epithelial cells that have several xenobiotic transporters on their apical and basolateral side, facilitating the excretion and absorption processes, as well as metabolizing enzymes (Morrissey et al. 2013; Secker et al. 2019). Thus, it is the segment of the nephron primarily exposed to drugs and their metabolites, and therefore the most likely section of the kidney to present with toxicity (Soo et al. 2018; Irvine et al. 2021). Despite advances in preclinical testing, unexpected toxicity during drug development accounts for about 30% of drug attrition (Guengerich 2011; Cook et al. 2014). Nephrotoxicity in particular is recognized only late in the drug development process, as it accounts for 2% of drug discontinuation in the preclinical stages and 19% in the clinic (Tiong et al. 2014). This leads to increased costs and time in developing pharmaceuticals (Guengerich 2011). Such low predictivity in preclinical settings has been attributed mainly to inter- and intra-species variability when performing in vivo animal trials and/or using in vitro models that do not mimic the patient’s in vivo state (Olson et al. 2000; Van Norman 2020; Madorran et al. 2020; Atkins et al. 2020).

A major factor often overlooked during in vitro nephrotoxicity testing, is the physiological oxygen tension of the kidney (4–14% O_2_). Routine cell culture under atmospheric oxygen (21% O_2_) can result in oxidative stress and respective adaptation processes, changing the cellular proteome, and introducing artefactual metabolization and transcription processes in the cell system (Kumar et al. 2016; Jagannathan et al. 2016; Timpano and Uniacke 2016; Mas-Bargues et al. 2019). Another important factor is that most in vitro models rely on healthy, fully differentiated cells and do not account for disease-associated effects (Benam et al. 2015; Wang et al. 2024). The latter would be crucial for acute kidney injury and chronic kidney disease, where greater cell proliferation rates are observed in an effort to restore organ homeostasis and thus functionality (Vogetseder et al. 2008; Witzgall 2008; Lee et al. 2021). Therefore, incorporating continuously proliferating cells into in vitro models may more precisely determine the susceptibility of these cells to add-on stress during pharmaceutical exposure and/or mimic the early processes involved during renal insult.

These limitations are apparent in chronic kidney diseases such as Autosomal Dominant Polycystic Kidney Disease (ADPKD). ADPKD is characterized by the progressive development of renal fluid-filled cysts, which expand and compress the surrounding healthy tissue, leading to local injury and fibrosis. Consequently, kidney function declines over time, resulting in end-stage renal disease in 50% of the cases (Torres et al. 2007; Bergmann et al. 2018). ADPKD is a heritable genetic disorder caused by mutations in the *PKD1* and *PKD2* genes, which encode for the PC1/PC2 polycystin complex. The latter modulates several signalling pathways of the renal epithelial cell, resulting in increased cAMP, upregulation of the vasopressin receptor 2 (V2R), activation of pro-proliferative pathways (MAPK/ERK, mTOR, β-catenin), transcription of cytokines, chemokines and growth factors and fluid secretion through CFTR (Torres and Harris 2014; Formica and Peters 2020). These changes disrupt the cell homeostasis and reprogram the metabolic pathways, driving the cell to the Warburg effect (Podrini et al. 2020; Haumann et al. 2020; Muto et al. 2022). Although numerous cellular molecules have been identified as druggable targets for ADPKD, only tolvaptan, a repurposed V2R antagonist, has been approved (a single dose of 60–120 mg results in C_max_ = 0.7 ± 0.2–1.5 ± 0.6 µM) for ADPKD treatment (Kim et al. 2011b). However, adverse off-target effects of tolvaptan, namely liver toxicity and polyuria, limit its use, and therefore, there is a need for developing drugs with higher efficacy and tolerability (Blair and Keating 2015; Torres et al. 2017).

Various molecules have been suggested as repurposed ADPKD drug candidates in the literature, as they have given pharmacologically promising results in in vitro cyst models or ameliorated the disease progression in PKD mice or were even tested clinically. Among these are the direct AMPK activator salicylic acid with an in vitro IC_50_ of 1.5 mM (Meyer et al. 2026), birinapant, a SMAC-mimetic that activates apoptosis, with an IC_50_ of 50 µM (Malas et al. 2020), bardoxolone methyl, an Nrf-2 activator and nuclear factor-κB inhibitor, which has shown in vitro to exert its pharmacological activity already at 200 nM (Huang et al. 2017), and rapamycin, an mTOR inhibitor, which attenuated cyst enlargement in vitro at a very low concentration (10 nM) (Booij et al. 2017; Malas et al. 2020). The reduction in cyst swelling in vitro or retardation of disease progression in PKD mice is certainly a promising step toward disease symptom amelioration. But the question still remains whether these compounds will directly affect the remaining healthy and functional renal proximal tubular epithelial cells (RPTEC). Additional toxicity could add to the genetically driven problem of the disease by reducing the remaining functionality of the kidney.

As current in vitro nephrotoxicity models do not account for physiological oxygen tension, cellular proliferation state, and disease-relevant conditions, the results derived from such experiments may produce unreliable results and thus limit their predictivity for assessing drug safety in ADPKD. Accordingly, we investigated the toxicity and metabolic effects of tolvaptan, salicylic acid, birinapant, bardoxolone methyl and rapamycin in human and mouse RPTEC cell lines exposed to atmospheric oxygen (21% O_2_ - AtmOx) and physiological oxygen (10% O_2_ - PhysOx) in a pharmacologically relevant range of concentrations. Differentiated and proliferating human (RPTEC/TERT1) and proliferating mouse (mProx24) cells served to mimic both the healthy and regenerative (after injury) states of the kidney (Berger et al. 2014; Eymael and Smeets 2016). We found that the toxicity depended on the proliferation state, species and oxygen partial pressure, and we observed that the cell models’ proliferation rate and metabolic activities are decisive factors for the outcome of in vitro toxicity testing. Further, and within the confines of the testing carried out, we identified salicylic acid and birinapant to have a suitable safety profile for ADPKD and provided a deeper understanding into the toxicity mechanism of tolvaptan in differentiated and proliferating cells.

## Materials and methods

### Chemicals

Tolvaptan (#S2593), bardoxolone methyl (#S8078) and rapamycin (#S1039) were purchased from Selleck Biotechnology GmbH, Germany. Birinapant (#HY-16591) was purchased from MedChemExpress (Hölzel Diagnostika Handels GmbH, Germany) and salicylic acid (#247588) from Sigma-Aldrich Chemie GmbH, Taufkirchen, Germany.

### Cell culture

Human TERT-immortalized renal proximal tubular epithelial cells (RPTEC/TERT1 (Secker et al. 2018a, b) were obtained from Evercyte GmbH (Vienna, Austria) and cultured in a 50:50 mix of DMEM no glucose (Fisher Scientific, #11520416) and Ham’s F-12 nutrient medium (VWR International, #L0135-500) supplemented with 2 mM GlutaMAX (Thermo Scientific, #35050061), 5 µg/mL insulin (Sigma, #I1882), 5 µg/mL transferrin (Sigma, #T2252), 5 ng/mL sodium selenite (Sigma, #S5261), 10 ng/mL epidermal growth factor (Sigma, #E9644), 36 ng/mL hydrocortisone (Sigma, #H0888), 1% penicillin-streptomycin, and 0.5% fetal bovine serum. Cells were passaged by trypsinization in a 1:3 ratio when confluent either in a standard incubator (~ 21% O_2_, 5% CO_2_, 37 °C – atmospheric oxygen; AtmOx) or in a SCI-tive Baker Ruskinn Low-Ox chamber (10% O_2_, 5% CO_2_, 37 °C, 75% humidity, medium equilibrated to incubator atmosphere overnight – physiological oxygen; PhysOx). RPTEC/TERT1 were seeded at a density of 60,000 cells/cm^2^ unless otherwise stated. Mouse renal proximal tubular epithelial cells (mProx24), SV40-large T transformed renal proximal tubule epithelial cells isolated from C57BL/6J adult mouse kidney (Takaya et al. 2003), were obtained from CMIC Group (Tokyo, Japan) and cultured in the same medium described for RPTEC/TERT1 either at AtmOx or PhysOx. The cells were passaged in a 1:5 ratio by trypsinization when confluent and were seeded at a density of 30,000 cells/cm^2^ unless otherwise stated.

### Growth curves

RPTEC/TERT1 and mProx24 cells were seeded at AtmOx or PhysOx in 12-well plates at a density of 10,000 cells/cm^2^. Every 1–2 days cells were trypsinized, scraped off and counted with the Countess™ II automated cell counter (Thermo Fisher Scientific, Germany) until they reached confluency (RPTEC/TERT1) or started dying (mProx24). All samples were prepared in three independent biological replicates, each consisting of two technical replicates. Population doubling times were calculated with a simple linear regression during the exponential growth phase.

### Glucose consumption and lactate production

RPTEC/TERT1 cells and mProx24 cells were seeded in 96-well plates at AtmOx or PhysOx. Differentiated RPTEC/TERT1 cells were cultured for 14 days with medium renewal every 2–3 days. On day 14, medium was renewed and 48 h later it was collected and saved for glucose and lactate quantification. Likewise, for proliferating RPTEC/TERT1 and mProx24 cells, medium was changed one day after seeding and collected 48 h later. Glucose consumption was analysed via an enzymatic reaction of glucose oxidase (GOD) and peroxidase (POD). 3.3 µL of the collected cell culture supernatant was diluted with 6.6 µL of 0.1 M sodium hydrogen phosphate (pH 6.5) and mixed with 90 µL reaction reagent (100 U/mL GOD, 0.25 U/mL POD and 1.5 mg/mL 2,2-azino-bis-[3-ethylbenzothiazoline-6-sulfonic acid] in 0.1 M sodium hydrogen phosphate). After incubating the plate for 40 min at room temperature, absorption was measured with a microplate reader (Infinite M200 or M200Pro, Tecan) at 420 nm. Glucose content was determined by using a standard curve (0–2 mM glucose). Cellular glucose consumption was calculated by subtracting the glucose in the supernatant from the starting amount of glucose in the medium. Lactate production was quantified as described previously (Limonciel et al. 2011), using a standard curve (0–25 mM lactate) with a spline fit and a microplate reader (Infinite M200 or M200Pro, Tecan) at 490 nm. All samples were prepared in three independent replicates, each consisting of three technical replicates. Results are presented for each cell type at AtmOx or PhysOx as the ratio of produced lactate to consumed glucose.

### Cell viability and cell death

RPTEC/TERT1 and mProx24 cells were seeded in 96-well plates and treated 14 days later for differentiated cells or the next day for proliferating cells with either tolvaptan (serial dilutions 0.78–100 µM) or salicylic acid (serial dilutions 0.078-10 mM) or birinapant (serial dilutions 0.78–100 µM) or bardoxolone methyl (serial dilutions 7.8–1000 nM) or rapamycin (serial dilutions 1.25–160 nM). For salicylic acid, birinapant, bardoxolone methyl and rapamycin, where data from in vitro studies were available (see Introduction), the concentration range was decided based on the effective concentrations and solubility of compounds. For tolvaptan, the common method of applying 20 to 200-fold higher concentrations than the C_max_ was used (Hengstler et al. 2020). The solvent control for tolvaptan, birinapant, bardoxolone methyl and rapamycin was 0.5% DMSO, while salicylic acid was directly dissolved in medium. For the co-treatment of tolvaptan and CCCP, RPTEC/TERT1 cultured at PhysOx were treated 24 h after seeding with either the solvent control (0.2% DMSO) or 50 µM tolvaptan or 1 µM CCCP or their combination for 90 min–24 h. Each experiment included cells treated with 0.1% Triton X-100 as positive control. All samples were prepared in three independent biological replicates, each consisting of three technical replicates. Cell viability and cell death were evaluated 48 h after treatment with the resazurin assay and the lactate dehydrogenase (LDH) assay, respectively. An aliquot of the cell culture supernatant was collected and saved for LDH quantification. A resazurin stock solution (110 µg/mL in PBS) was diluted 1:10 in cell culture medium and added to the cell plate. The plate was subsequently incubated for 1–2 h at AtmOx or PhysOx upon which the supernatant was transferred to a black microplate and its fluorescence (Ex: 530 nm / Em: 590 nm) was measured using a microplate reader (Infinite M200, Tecan). Viability was presented as percent of the solvent control.

LDH leakage (loss of plasma membrane integrity) was used as a marker of cytotoxicity, whereby 10 µL or 20 µL of cell culture supernatant was mixed with 190 µL or 180 µL (for differentiated or proliferating cells, respectively) of LDH reaction solution (1.25 mM NADH and 3.1 mM sodium pyruvate in 25 mM potassium phosphate buffer pH 7.5). The absorption of NADH at 37 °C was recorded every minute for 31 cycles with a microplate reader (Infinite M200 or M200Pro, Tecan) at 340 nm. The slope of the linear regression of the absorption was calculated, which directly corresponded to the amount of LDH released into the cell culture supernatant. Cells were finally lysed with 0.1% Triton X-100 in PBS to quantify the intracellular LDH content. The results were presented as percent cell death calculated by dividing the extracellular LDH activity by the sum of extracellular and intracellular activities.

Based on the generated cell viability and cell death curves, the Lowest Effective Concentrations (LoECs), the Effective Concentration 20% (EC_20_) and the slopes of the curves were calculated in GraphPad Prism 9.0.0 to facilitate data interpretation and statistical analysis.

### Metabolic ATP production rates

The evaluation of the ATP production rates of RPTEC/TERT1 at PhysOx was performed as previously described (Meyer et al. 2026) with a Seahorse XFe24 Analyzer (Agilent, Germany) installed inside a Low-Ox chamber (10% O_2_, 0.1% CO_2_, 37 °C). Cells were seeded 7 days prior to the experiment in Seahorse XF24 culture plates at a density of 140,000 cells/cm^2^ in their standard medium at PhysOx. Ninety minutes before the start of the assay, cells were exposed to Seahorse XF DMEM medium pH 7.4 supplemented with 5 mM glucose, 0.5 mM pyruvate and 2.5 mM glutamine, containing the solvent control or the test compounds. 0.2% DMSO served as the control for tolvaptan (25 and 100 µM), birinapant (4, 20 and 100 µM), bardoxolone methyl (40, 200 and 1000 nM) and rapamycin (10 and 100 nM), while salicylic acid (0.1, 1 and 10 mM) was dissolved in Seahorse medium. Since the pH of 10 mM salicylic acid in Seahorse medium was 5.0, it was adjusted to approximately 7.4 by adding NaOH. Cells were analysed with the XF Real-Time ATP Rate Assay Kit according to the manufacturer’s instructions. Briefly, right before the start of the assay, the medium was renewed exactly as described above. Oxygen Consumption Rate (OCR) and Extracellular Acidification Rate (ECAR) were measured basally, and after the injection of first 1.5 µM oligomycin, and then 0.5 µM rotenone and 0.5 µM antimycin A. At the end of the assay, nuclei were stained with 2.5 µM 4′,6-diamidino-2-phenylindole (DAPI) and counted with a Cellomics Array-Scan VTI HCS Reader (Thermo Scientific, Germany). Data was normalized per 10,000 cells and OCR, ECAR and ATP rates values were exported from the Seahorse Wave Desktop Software (Agilent, Santa Clara) to excel. All samples were prepared in three independent biological replicates, each consisting of four technical replicates.

### Reactive oxygen species (ROS) and superoxide production

RPTEC/TERT1 cells were seeded in clear bottom black 96-well microplates at a density of 140,000 cells/cm^2^ at PhysOx or AtmOx. Cells were treated 7 days later with either tolvaptan (5, 25, 50 and 100 µM), salicylic acid (0.1, 1, 5 and 10 mM), birinapant (4, 20, 50 and 100 µM), bardoxolone methyl (40, 200, 500 and 1000 nM), rapamycin (1, 10, 50 and 100 nM), CCCP (0.5, 1, 5 and 50 µM), NAC (0.1, 1, 5 and 10 mM), or pyocyanin (500 µM). Each experiment included the respective solvent control, i.e. 0.2% DMSO for tolvaptan, birinapant, bardoxolone methyl and CCCP, 2% water for NAC, 5% DMF for pyocyanin and medium without phenol red for salicylic acid. Treatment times were 90 min for tolvaptan, salicylic acid, birinapant, bardoxolone methyl and rapamycin, 15 min for CCCP, 60 min for NAC and 20 min for pyocyanin. All samples were prepared in three independent biological replicates, each consisting of six technical replicates. ROS/Superoxide production was evaluated with a ROS-ID^®^ Total ROS/Superoxide Detection Kit (Enzo, #ENZ-51010) using a fluorescence microplate reader (Infinite M200, Tecan), according to the manufacturer’s instructions. Results were presented as fold-change of fluorescence compared to the respective solvent control.

### Mitochondrial membrane potential (MMP)

MMP was assessed in RPTEC/TERT1 at PhysOx following the same seeding and treatment protocol as described for ROS/Superoxide Production by tetramethylrhodamine ethyl ester (TMRE) staining. TMRE is a cationic fluorescent dye that penetrates the cell membrane and is sequestered by active mitochondria, directly corresponding to the MMP (Crowley et al. 2016). For the co-treatment of tolvaptan and CCCP, RPTEC/TERT1 cultured at PhysOx were treated 24 h after seeding with either the solvent control (0.2% DMSO) or 50 µM tolvaptan or 1 µM CCCP or their combination for 90 min. All samples were prepared in three independent biological replicates, each consisting of six technical replicates. At the end of the treatment period, cells were stained with 200 nM TMRE in cell culture medium, incubated at 37 °C for 20 min, washed 3 times with PBS and fluorescence (Ex: 550 nm / Em: 580 nm) was quantified with a plate reader (Infinite M200, Tecan). Results were presented as fold-change of fluorescence compared to the respective solvent control.

### Cell cycle analysis

RPTEC/TERT1 and mProx24 were seeded in 60 mm petri dishes at PhysOx. The cells were treated 7 days later for differentiated RPTEC/TERT1 (seeding density 140,000 cells/cm^2^) and 24 h later for proliferating RPTEC/TERT1 and mProx24 with either the solvent control (0.2% DMSO) or tolvaptan (5 and 25 µM) for 24 h. The cells were then washed with PBS, trypsinized, collected and centrifuged for 5 min at 500 rcf. The supernatant was discarded and the cells were resuspended in PBS and centrifuged for 5 min at 500 rcf. After discarding the supernatant, the cells were resuspended in 500 µL of PBS. An equal volume (500 µL) of methanol: acetone (4:1, v/v) was then added, and the cells were incubated at 4 °C for 30 min. The fixed cells were diluted with PBS and centrifuged for 5 min at 500 rcf. After centrifugation, the supernatant was discarded and the cell pellet was resuspended in 250 µL propidium iodide (PI) staining solution (100 µg/mL). To prevent RNA-associated fluorescence, RNase A was added at a final concentration of 100 µg/mL. Cells were incubated for 4 h at RT in the dark. Subsequently, samples were analysed using a BD FACSLyric flow cytometer and PI signals were measured using excitation by the 488 nm laser and a 586/42, 560LP filter set. A minimum of 10,000 events were acquired per sample, three independent biological replicates were prepared. Cell cycle distribution and DNA content histograms were generated and analysed using FlowJo software (version 10.4.2) (Watson et al. 1987). Results were presented as the percentage of the cells in the different phases of the cell cycle and based on this, the G2-to-G1 ratio was also calculated.

### Determination of 8-oxo-7,8-dihydro-2′-deoxyguanosine (8-oxo-dG)

#### DNA isolation and digestion

RPTEC/TERT1 were seeded in 100 mm petri dishes at PhysOx. The cells were treated 7 days later for differentiated RPTEC/TERT1 (seeding density 140,000 cells/cm^2^) and 24 h later for proliferating RPTEC/TERT1 with either the solvent control (0.2% DMSO) or tolvaptan (100 µM) for 90 min. As a positive control, cells were treated with KBrO_3_ (5 mM) and the respective solvent control (media) for 2 h. After the treatment period, the DNA was isolated and digested according to a recent protocol with slight modifications (Gerdemann et al. 2025). The cells were washed twice with PBS, lysed with 500 µL lysis buffer (20 mM tris(hydroxymethyl)aminomethane, 250 mM NaCl, 25 mM EDTA, 0.5% SDS, pH 8.5) and collected in tubes. Then 50 µg RNase A was added and the samples were shaken gently for 30 min at 37 °C, followed by incubation with 40 µg proteinase K overnight at 4 °C. The remaining proteins were precipitated with 300 µL potassium acetate (5 M, pH 7) and the samples were centrifuged (14,800 rcf, 4 °C) until the supernatant could be clearly separated from the pellet. DNA was precipitated by adding 1 mL isopropanol and gently inverting the tubes. The samples were centrifuged (14,800 rcf, 4 °C, 30 min), the supernatant was discarded and 500 µL 70% ethanol was added. After centrifuging the samples (14,800 rcf, 4 °C, 15 min) and discarding the supernatant, the DNA was dried under vacuum and dissolved in 100 µL digestion buffer (10 mM tris(hydroxymethyl)aminomethane hydrochloride, 5 mM MgCl_2_, pH 7). For the digestion, 2 U DNase, 100 mU phosphodiesterase and 500 mU alkaline phosphatase were added and the samples were incubated overnight at 37 °C. Afterwards, 300 µL acetonitrile were added to precipitate digesting enzymes. The supernatant was dried under vacuum and redissolved in 100 µL water. An aliquot was diluted twice by a factor of 100 to enable data normalization to deoxyguanosine (dG) content.

#### LC–MS/MS analysis

DNA lysates were separated on a Nucleodur C18 Pyramid column (Macherey-Nagel, Düren, Germany, 150 × 2 mm, 3 μm) using a gradient of acetonitrile and water both supplemented with 0.1% formic acid. Both solvents were used in MS grade. The flow rate was set to 0.5 mL/min and the gradient started at 100% aqueous solvent. After two minutes, organic part was increased to 95% within 8 min followed by column flushing and re-equilibration. The column oven had a temperature of 40 °C and the autosampler was cooled to 15 °C. Chromatographic separation was performed using an Agilent Infinity II Bio HPLC system (Waldbronn, Germany) in combination with a QTRAP 7500 mass spectrometer (Sciex, Darmstadt, Germany). The mass spectrometer was operated in positive multiple reaction monitoring mode and the following transitions were used for quantification of 8-oxo-dG and dG respectively: 284.1/168.1 (CE 20 V), 268.1/152.1 (CE 15 V). Further mass spectrometric parameters were provided in the supplementary material (Table 1S). Results were presented as ratio of 8-oxo-dG to dG.

### Immunocytochemistry (ICC) for γ-H2AX detection

RPTEC/TERT1 and mProx24 were seeded on round 12 mm glass cover slips inside 24-well plates at PhysOx. The cells were treated 7 days later for differentiated RPTEC/TERT1 (seeding density 140,000 cells/cm^2^) and 24 h later for proliferating RPTEC/TERT1 and mProx24 with either the solvent control (0.2% DMSO) or tolvaptan (5, 25 and 100 µM) or bardoxolone methyl (1000 nM) for 90 min. As a positive control, cells were treated with H_2_O_2_ (50 and 250 µM) and the respective solvent control (media) for 20 min. Media was then aspirated and the cells were fixed with 4% PFA in PBS for 25 min at RT, washed three times with PBS, permeabilized with 0.1% Triton-X 100 in PBS for 10 min at RT, washed twice with PBS and blocked with 1% BSA in TBST (Tris-Buffered Saline with Tween-20) for 1 h at RT. The fixed cells were incubated with an anti-γ-H2AX primary antibody (Anti-gamma H2A.X (phospho S139) antibody, Abcam #ab11174, host species rabbit, lot 1107510-9) diluted 1:500 in the blocking buffer overnight at 4 °C. The cover slips were then washed three times with the blocking buffer and incubated with the secondary antibody (Alexa Fluor^®^ 647 goat anti-rabbit IgG (H + L), LifeTechnology #A-21244, lot 1386544) diluted 1:1000 in the blocking buffer for 2 h at RT in the dark. The secondary antibody was removed and the cells were incubated with 2.5 µM DAPI (Hoechst 33342) in PBS for 10 min at RT in the dark. Finally, cells were washed three times with PBS, transferred to glass slides, mounted with fluorescence mounting medium and sealed with nail polish. All samples were prepared in three independent replicates. Imaging was performed by capturing three images per sample, each from a different region of the cover slip, using a Zeiss LSM 700-point laser scanning confocal microscope (Bio-Imaging Centre, University of Konstanz). Mean fluorescence intensity of γ-H2AX of each image was calculated with ImageJ (Schneider et al. 2012). Data was presented as representative captured images and mean γ-H2AX intensity per image.

### Fluorimetric detection of alkaline DNA unwinding (FADU)

RPTEC/TERT1 and mProx24 were seeded in 96-well plates at PhysOx. The cells were treated 7 days later for differentiated RPTEC/TERT1 (seeding density 140,000 cells/cm^2^) and 24 h later for proliferating RPTEC/TERT1 and mProx24 with either the solvent control (0.2% DMSO) or tolvaptan (5, 25 and 100 µM) or bardoxolone methyl (200, 500 and 1000 nM) for 90 min. As a positive control, cells were treated with H_2_O_2_ (50, 250 and 500 µM) and the respective solvent control (media) for 20 min. Then the media was aspirated, the plates were washed twice with PBS and directly transferred to a TECAN Genesis RSP 100 liquid handling device (LHD) from TECAN AG (Hombrechtikon, Switzerland), controlled by GEMINI V4.0 software from TECAN software GmbH (Berlin, Germany). FADU was performed exactly as described by Moreno-Villanueva et al. 2009. Immediately after the addition of SybrGreen, the fluorescence was measured (Ex: 492 nm / Em: 520 nm) with a microplate reader (Infinite M200, Tecan). All samples were prepared in three independent replicates, each consisting of six technical replicates. Fluorescence values of the wells where DNA unwinding had been induced (P samples) were divided by the values of the respective wells where DNA unwinding had not been induced (T samples- total DNA content) and the ratio was multiplied by 100, providing the percentage of double-stranded DNA remaining in the lysate after the alkaline unwinding, which originates from DNA strand breaks. High P values are therefore indicative of low numbers of strand breaks and vice versa.

### Statistics

Data was plotted and statistically analysed using GraphPad Prism 9.0.0 and was presented as the mean of three independent replicates ± SEM, unless otherwise stated. Results of statistical analyses were noted in the description of each figure, whereby the significance thresholds were defined at *p* < 0.05, *p* < 0.01, *p* < 0.001 or *p* < 0.0001.

## Results

### Cell proliferation and metabolic features of murine and human RPTEC at AtmOx and PhysOx

**Fig. 1:**
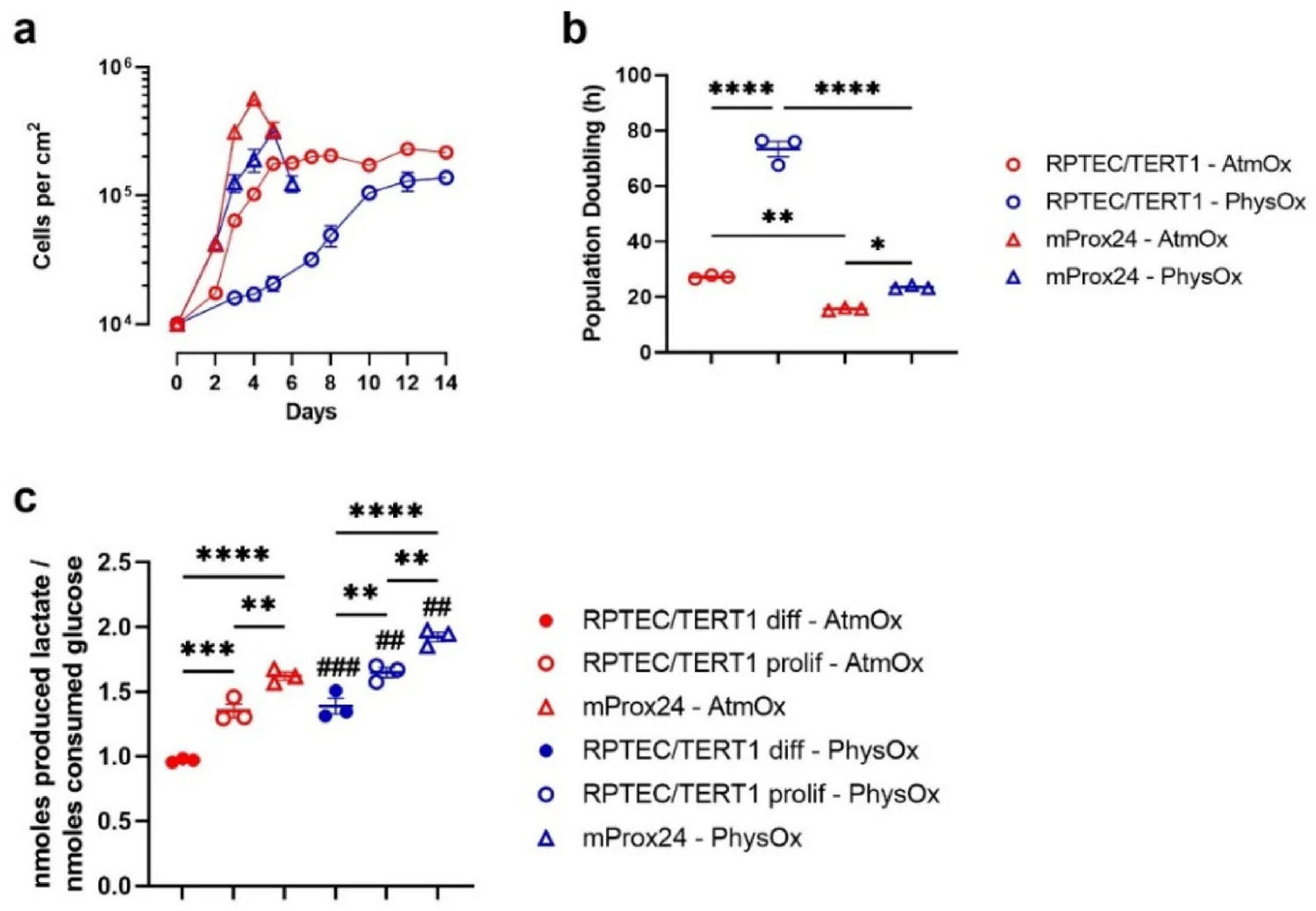
**a** Proliferation curves and **b** population doubling times of RPTEC/TERT1 and mProx24 at AtmOx and PhysOx. **c** Lactate to glucose ratio of differentiated and proliferating RPTEC/TERT1 and mProx24 at AtmOx and PhysOx. (a) Each point represents the mean of three independent biological replicates ± SEM. (b and c) Each point represents one independent replicate and each line the mean all replicates ± SEM. *P < 0.05, ## or **P < 0.01, ### or ***P < 0.001, ****P < 0.0001 one-way ANOVA followed by Šídák’s multiple comparisons test. The symbol # indicates statistical significance between the same cell types at AtmOx and PhysOx

As an initial characterization, cell proliferation and metabolic features of proliferating murine and differentiated and proliferating human RPTEC were determined at AtmOx and PhysOx. As depicted in Fig. 1a, the growth curves of proliferating human (RPTEC/TERT1) and mouse (mProx24) renal proximal tubular cells at AtmOx and PhysOx presented with slower growth at PhysOx. Indeed, the difference in growth at different O_2_ tensions was much more succinct in RPTEC/TERT1 than in mProx24 (Fig. 1b). RPTEC/TERT1 at PhysOx presented with a doubling time of ~ 70 h, while at AtmOx a doubling time of ~ 28 h was recorded (2.5-fold faster). The population doubling of mProx24 cells at AtmOx was ~ 16 h and at PhysOx ~ 24 h (1.5-fold slower). Moreover, the contact-inhibited h-TERT immortalized human cells created a stable monolayer after reaching confluency, whereas the SV40 immortalized murine cells started dying once they reached confluency.

As expected (Chandel 2021), proliferating cells were much more glycolytic than the corresponding differentiated RPTEC/TERT1 cells (Fig. 1c). Corresponding to the higher proliferation rate (Fig. 1a and b), mProx24 also were significantly more glycolytic than proliferating or differentiated RPTEC/TERT1 cells. Moreover, physiological oxygen conditions significantly exacerbated the shift towards glycolysis in all cells, although the proliferation rate decreased decisively in case of RPTEC/TERT1 and moderately in case of mProx24. The latter was interpreted as an adaptive response to PhysOx, as observed earlier (Piossek et al. 2022), whereby a high amount of lactate appeared also to be funnelled into other anabolic processes (Chandel 2021) typical for proximal tubule epithelial cells.

### Toxicity of ADPKD drugs is dependent on proliferation state, species and oxygen partial pressure

As a first point of orientation, toxicity curves, using cell viability and cell death as endpoints, were established for all compounds in RPTEC/TERT1 (differentiated and proliferating cells) and mProx24 (proliferating cells). When cultured for several days, RPTEC/TERT1 cells have been described to undergo a maturation process that results in a monolayer of functionally differentiated cobblestone-shaped epithelial cells, mimicking the healthy proximal tubule (Aschauer et al. 2013; Secker et al. 2018b; Piossek et al. 2022). Proliferating RPTEC/TERT1 or mProx24 cells were used as a model of the robust proliferation that occurs during renal insult (Berger et al. 2014; Kusaba and Humphreys 2014; Eymael and Smeets 2016; Secker et al. 2018a). The selected range of concentrations reflects those reported in the literature to be pharmacologically effective in preclinical or clinical settings, as stated in the Introduction.

**Fig. 2:**
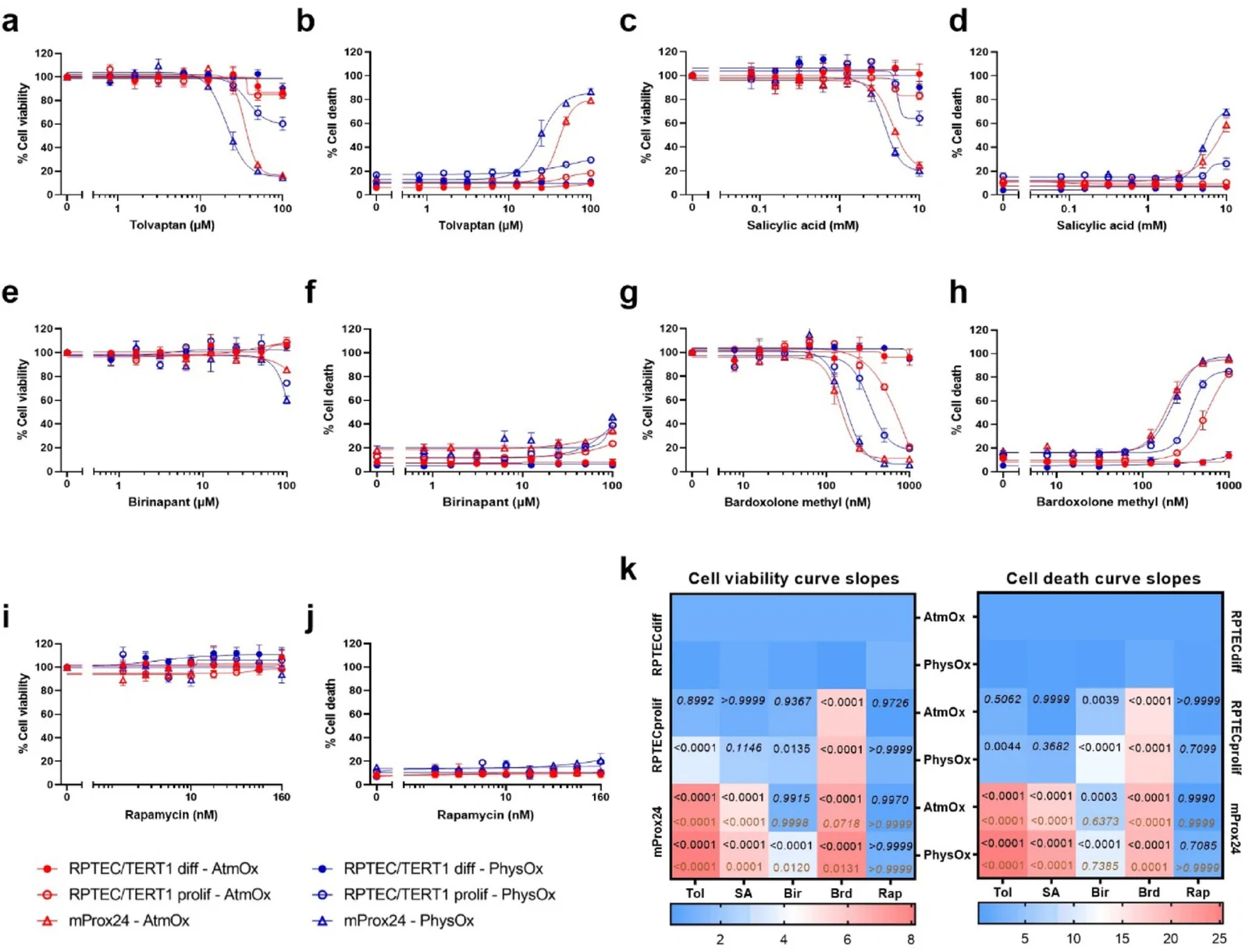
**a**–**j** Cell viability (resazurin assay) and cell death (LDH leakage assay) toxicity concentration-response curves of differentiated RPTEC/TERT1, proliferating RPTEC/TERT1 and proliferating mProx24 at AtmOx and PhysOx 48 h after exposure to tolvaptan (**a**, **b**), salicylic acid (**c**, **d**), birinapant (**e**, **f**), bardoxolone methyl (**g**, **h**) and rapamycin (**i**, **j**). Each point represents the mean of three independent biological replicates ± SEM. **k** Fold-change of the respective cell viability (left) and cell death (right) toxicity curve slopes of tolvaptan (Tol), salicylic acid (SA), birinapant (Bir), bardoxolone methyl (Brd) and rapamycin (Rap) relative to differentiated RPTEC/TERT1 at AtmOx (bar below heat-map). Numbers in the heat-map indicate (black: *p*-values between differentiated RPTEC/TERT1 and proliferating RPTEC/TERT1 or mProx24 at AtmOx or PhysOx; brown: p-values between proliferating RPTEC/TERT1 and mProx24 at AtmOx or PhysOx). *P*-values were calculated using a one-way ANOVA followed by Šídák’s multiple comparisons test. Italicized values are above the threshold of significance (*p* < 0.05).

Exposure to tolvaptan, salicylic acid, birinapant and bardoxolone methyl provided for a comparable toxicity i.e. concentration-response curve for cell viability and cell death, irrespective of the O_2_ tension (PhysOx or AtmOx) used (Fig. 2a–j), while no toxicity was observed for rapamycin. Distinct differences were observed in the toxicity curves when comparing the different cell types at AtmOx or PhysOx. Indeed, proliferating mProx24 appeared most sensitive, followed by proliferating RPTEC/TERT1, whereby differentiated RPTEC/TERT1 were the least sensitive. The latter observations were confirmed by calculating the slopes of the toxicity curves of each compound (Table 2S). The heat maps in Fig. 2k represent the fold-change of all concentration-response curve slopes at AtmOx and PhysOx of all cells, when compared to the differentiated RPTEC/TERT1 at AtmOx (absolute values of the slopes are presented in Table 2S). For each drug (except rapamycin), the slope became steeper (indicating higher toxicity) when moving from differentiated RPTEC/TERT1 to proliferating RPTEC/TERT1 to mProx24 at AtmOx or PhysOx both for cell viability and cell death (Fig. 2k).

Lowest Effect Concentrations (LoEC) were calculated for each compound and testing condition (Table 3S). No toxicity, and thus no LoEC, could be determined for any of the tested compounds in differentiated RPTEC/TERT1 at both AtmOx and PhysOx, except for tolvaptan (LoEC _viability_ = 100 µM) at PhysOx. More importantly, proliferating mProx24 cells appeared to be more sensitive than proliferating RPTEC/TERT1 cells, as shown above.

Although the calculation of LoEC provided an estimation of the toxicity expected and for most compounds confirmed the general trend, it did not provide for deeper insight into the concentration-response differences observed between AtmOx and PhysOx. Hence, the Effective Concentration 20 (EC_20_) was calculated (Table 1) for proliferating RPTEC/TERT1 and mProx24, for the drugs that showed toxicity at either O_2_ tension. In proliferating RPTEC/TERT1 the EC_20_ for cell viability of tolvaptan, salicylic acid, birinapant and bardoxolone methyl was up to 2.5-fold higher at AtmOx than at PhysOx. Cell death EC_20_ values had generally the same trend. In the mouse cells, the EC_20_ of tolvaptan, salicylic acid and birinapant was up to 2-fold higher at AtmOx than at PhysOx. It is apparent that in many cases, the calculated viability values were equal to or lower than those for cell death. The latter is most likely due to the fact that cell viability determination employed here used reduction of mitochondrial activity, whereas to detect changes in cell death, LDH has to be released into supernatant, thus requiring disintegration of cell membrane integrity. This most likely would occur at higher compound concentrations.

**Table 1:** Effective concentration 20% (EC_20_) of cell viability and cell death curves of tolvaptan (Tol), salicylic acid (SA), birinapant (Bir), and bardoxolone methyl (Brd) in proliferating RPTEC/TERT1 and mProx24 at AtmOx and PhysOx

Cell type	RPTEC/TERT1 proliferating				mProx24 proliferating			
Assay	Cell viability		Cell death		Cell viability		Cell death	
EC20	AtmOx	PhysOx	AtmOx	PhysOx	AtmOx	PhysOx	AtmOx	PhysOx
Tol	> 100 µM	41.0 µM	> 100 µM	> 100 µM	28.6 µM	13.4 µM	28.7 µM	15.4 µM
SA	> 10 mM	7.4 mM	> 10 mM	6.0 mM	3.2 mM	2.6 mM	7.5 mM	5.1 mM
Bir	> 100 µM	98.1 µM	> 100 µM	73.4 µM	> 100 µM	80.5 µM	> 100 µM	78.8 µM
Brd	382.7 nM	221.3 nM	369.2 nM	249.6 nM	108.4 nM	121.9 nM	131.4 nM	146.9 nM

The above observations suggest that tolvaptan, salicylic acid, birinapant and bardoxolone methyl result in greater toxicity in proliferating than in differentiated renal proximal tubular epithelial cells, with proliferating cells being more sensitive at PhysOx than at AtmOx. Moreover, the comparison of murine and human proliferating cells suggested much higher sensitivity of murine RPTEC to these compounds, underscoring the issue of species differences also of in vitro testing and thus in new approach methods (Punt et al. 2020; Schmeisser et al. 2023; Hartung and Kleinstreuer 2025).

### Drug-induced changes in cell metabolism

Since proliferative *PKD1*-deficient cells display a Warburg-like metabolic reprogramming (increased glucose consumption, lactate production, glutamine usage, ATP levels, fatty acid biosynthesis, and oxidative stress) (Podrini et al. 2018, 2020; Haumann et al. 2020), it was important to determine whether the observed toxicity was related to and would have an add-on effect on mitochondrial dysfunction. Therefore, changes of ATP metabolic rates, ROS/Superoxide production and Mitochondrial Membrane Potential (MMP) were investigated after drug treatment on differentiated RPTEC/TERT1. Cells cultured at AtmOx have been described to experience a high oxygenation environment with excessive ROS and oxidative stress (Kumar et al. 2016; Jagannathan et al. 2016). Accordingly, considering that physiological oxygen cultures more closely represent the in vivo state, we investigated more in depth the underlying mechanisms of the observed toxicity at PhysOx. To detect potential differences in ROS generation, a selected number of concentrations and compounds were also tested at AtmOx.

**Fig. 3:**
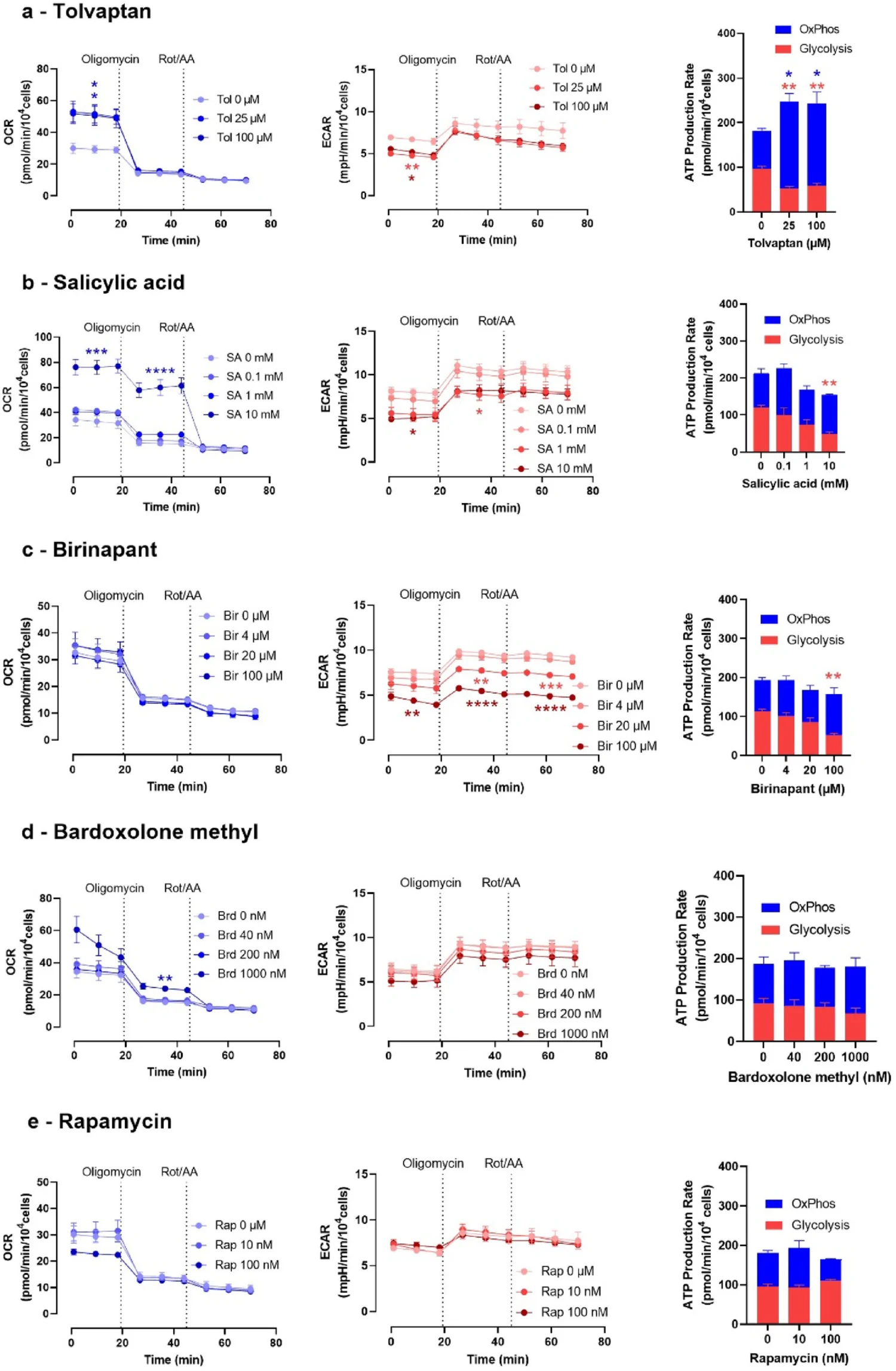
Oxygen Consumption Rate (OCR), Extra-Cellular Acidification Rate (ECAR) and total ATP Production Rate of differentiated RPTEC/TERT1 at PhysOx after 90 min pre-treatment with tolvaptan (Tol—**a**), salicylic acid (SA - **b**), birinapant (Bir—**c**), bardoxolone methyl (Brd— **d**) and rapamycin (Rap—**e**). Each point represents the mean of three independent replicates ± SEM. *P < 0.05, **P < 0.01, ***P < 0.001, ****P < 0.0001 calculated by one-way ANOVA followed by Dunnett’s multiple comparisons test relative to the solvent control. Figures b-d are part of Meyer et al. (2026) and were included for comparative reasons

**Fig. 4:**
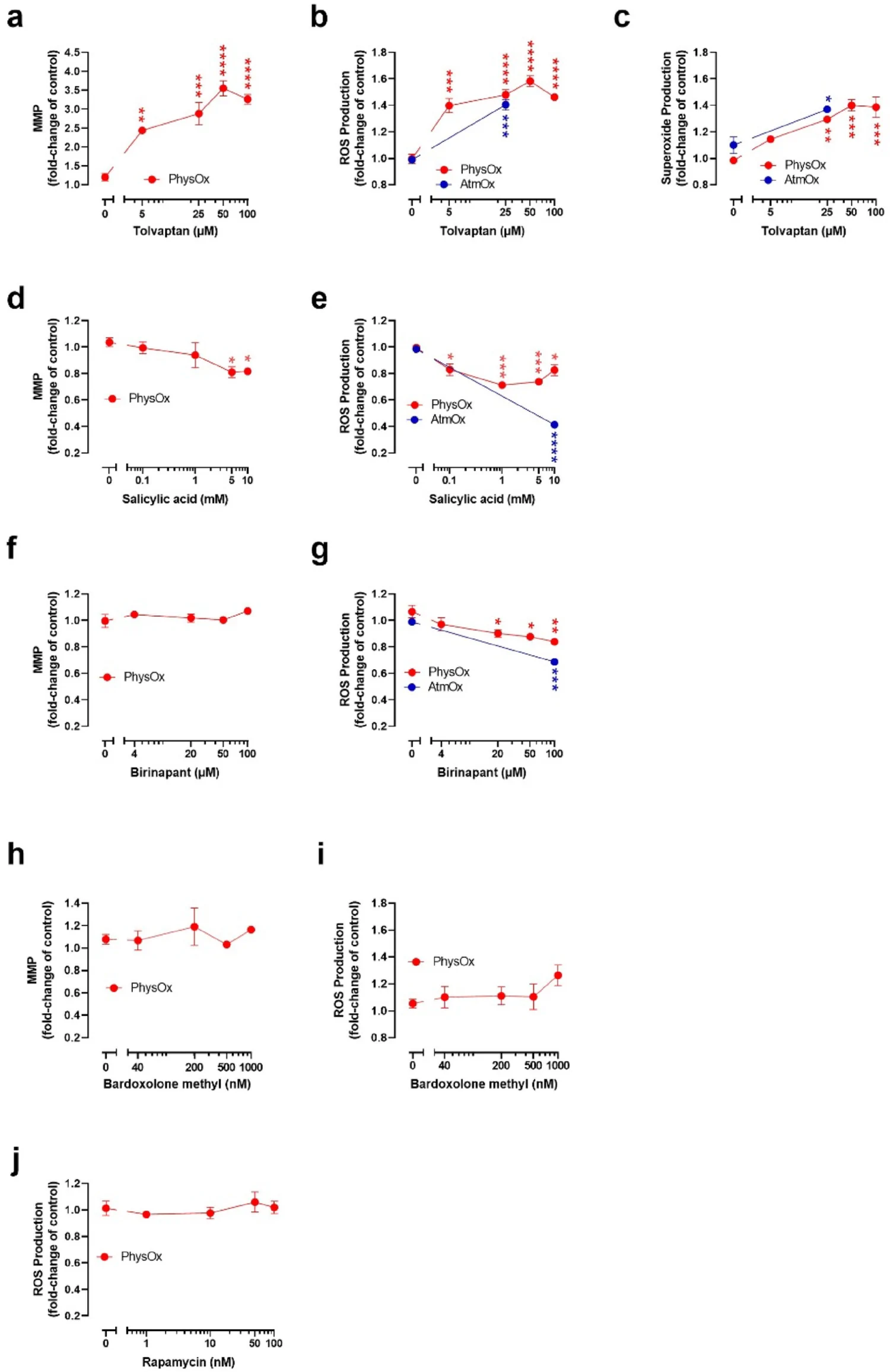
Fold-change relative to the solvent control of mitochondrial membrane potential (MMP) and ROS/superoxide production of differentiated RPTEC/TERT1 at PhysOx and AtmOx after 90 min treatment with tolvaptan (**a**, **b**, **c**), salicylic acid (**d**, **e**), birinapant (**f**, **g**), bardoxolone methyl (**h**, **i**) and rapamycin (**j**). Each point represents the mean of three independent replicates ± SEM. *P < 0.05, **P < 0.01, ***P < 0.001, ****P < 0.0001 calculated by one-way ANOVA followed by Dunnett’s multiple comparisons test relative to the solvent control (when more than two groups were compared) or unpaired t-tests (when two groups were compared)

Tolvaptan (Fig. 3a) treatment resulted in an increased Oxygen Consumption Rate (OCR) and total ATP production, while the Extracellular Acidification Rate (ECAR) was decreased. It also resulted in increased MMP by up to 3-fold and ROS and Superoxide production by up to 1.5-fold when compared to the solvent control (Fig. 4a-c). A saturation effect was observed at concentrations ≥ 25 µM. All data suggested a direct or indirect interaction of tolvaptan with oxidative phosphorylation pathways, shifting the metabolism away from glycolysis, by increasing oxygen consumption, which led to a more active mitochondrial electron transport chain, which in turn increased MMP and ATP production. Elevated ROS and Superoxide were most likely by-products of the enhanced mitochondrial function of the complex I and III (Chen et al. 2003; Hirst et al. 2008; Bleier and Dröse 2013; Nolfi-Donegan et al. 2020). The latter appeared to occur independent of whether RPTEC/TERT1 were cultured at PhysOx or AtmOx, as no differences in ROS or Superoxide generated were observed in cells exposed to 25 µM tolvaptan at AtmOx and PhysOx (Fig. 4b and c). The hyperpolarization of the mitochondrial membrane caused by tolvaptan was reversible when proliferating RPTEC/TERT1 at PhysOx were co-treated with 50 µM tolvaptan and 1 µM CCCP (mitochondrial uncoupler) (Demine et al. 2019) for 90 min without any concurrent reduction in cell viability (Fig. S1a and b). CCCP also partially rescued the reduction in cell viability induced by tolvaptan after 24 h (approximately 15% increase, Fig. S1c).

In contrast, salicylic acid, albeit significantly increasing OCR, significantly decreased ECAR and total ATP production in a concentration-dependent manner (Fig. 3b). The concurrently reduced MMP and ROS production (Fig. 4d and e), resembled the activity observed for CCCP (Fig. S2a and b), and thus could be indicative of uncoupling activity (Demine et al. 2019), which has been reported for salicylic acid-treated mitochondria isolated from plants and rat liver (Thompkins and Lee 1969; Norman et al. 2004). It is interesting to note that at AtmOx 10 mM salicylic acid treatment reduced ROS to a much larger extent than at PhysOx (Fig. 4e). The latter was also observed for the concurrent uncoupling control CCCP (Fig. S2b), thus suggesting that the mitochondrial uncoupling by salicylic acid and CCCP was more efficient at AtmOx than at PhysOx.

Birinapant did not change OCR, but decreased ECAR in a concentration-dependent manner, suggestive of reduced glycolysis (Fig. 3c). Furthermore, birinapant reduced ROS production in a concentration-dependent manner, while it did not affect MMP (Fig. 4f and g). The latter was comparable to NAC (Fig. S2c and d) (Pedre et al. 2021), suggesting ROS scavenging activity of birinapant (Rafat et al. 2023). The ROS scavenging activity of NAC and birinapant were observed to occur in a similar fashion and intensity at PhysOx and AtmOx (Fig. S2d and 4 g).

Bardoxolone methyl increased OCR at 1000 nM, while ECAR was unaffected (Fig. 3d), implying a metabolic shift towards oxidative phosphorylation. Similarly, ROS was increased by 1.2-fold (not significant) only at the highest concentration (1000 nM) and the MMP was unchanged (Fig. 4h and i), suggesting that the high toxicity seen (Fig. 2g and h) was not related to oxidative stress.

Finally, rapamycin which induced no toxicity in any of the murine or human RPTEC, did not induce any determinable metabolic changes (Figs. 3e and 4j).

### Tolvaptan triggered DNA oxidative damage

The mitochondrial oxidative stress triggered by tolvaptan raised the question whether it can also induce DNA damage. It is known that prolonged exposure to elevated ROS and superoxide levels can directly oxidize, among other molecules, the nucleosides of DNA, e.g., forming 8-oxo-7,8-dihydro-2′-deoxyguanosine (8-oxo-dG) in place of deoxyguanosine (dG) (Gorini et al. 2021; Huang et al. 2021; Hahm et al. 2022). These DNA lesions, if left unrepaired, lead to transversions, genomic instability and double-strand breaks (DSBs), and DNA damage response (DDR) activation, which includes cell cycle arrest and programmed cell death (Boonstra and Post 2004; Jackson and Bartek 2009; Matic 2018; Safwat et al. 2022; Mackova et al. 2024). Hence, since tolvaptan treatment led to mitochondrial toxicity, the potential of also initiating DDR was investigated.

**Fig. 5:**
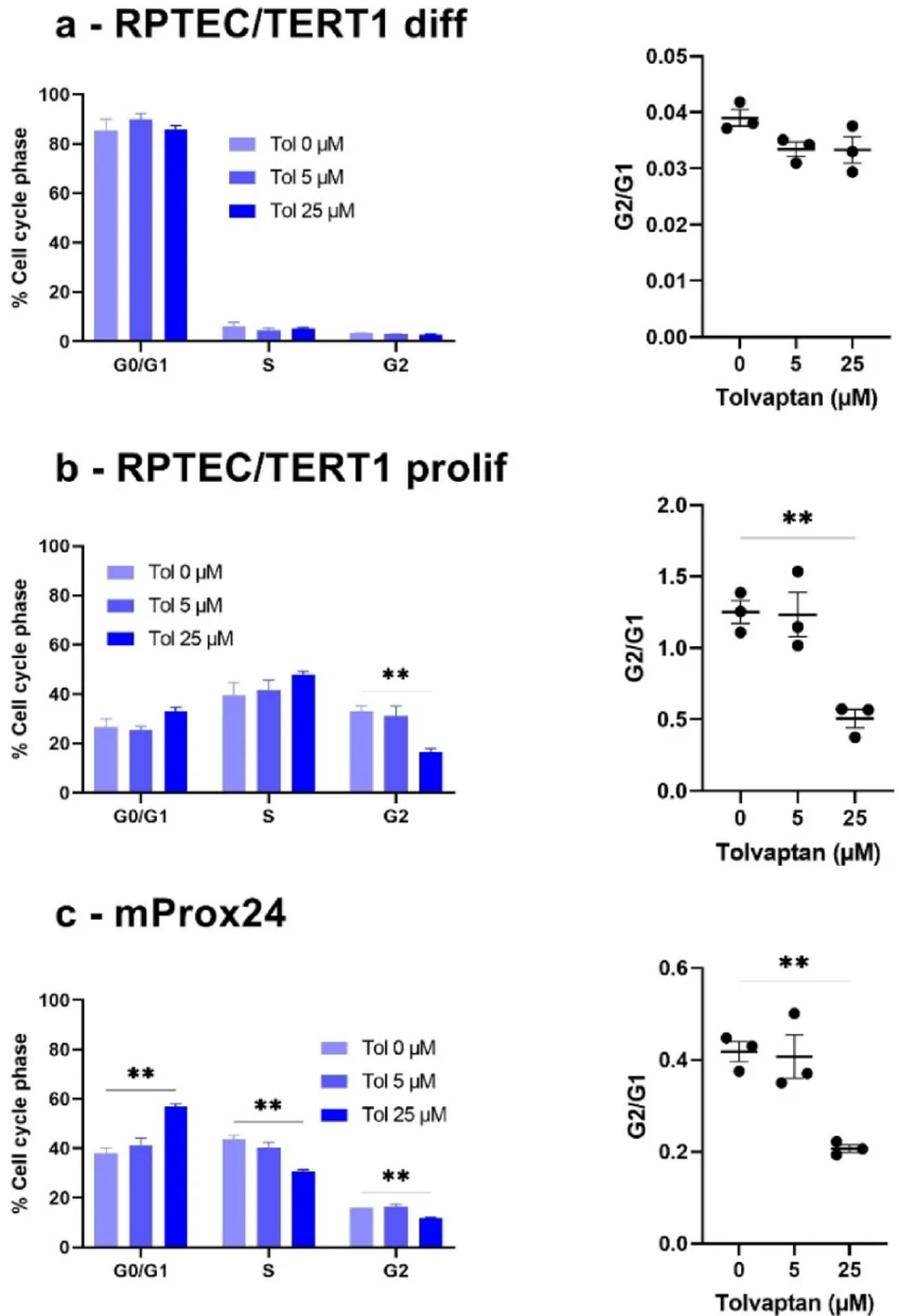
Distribution of cells in the different phases of the cell cycle and G2-to-G1 ratio of differentiated (**a**) and proliferating (**b**) RPTEC/TERT1 and mProx24 (**c**) after 24 h treatment at PhysOx with 0, 5 and 25 µM tolvaptan (Tol). Each point represents the mean of three independent replicates (% of cells in the cell cycle phases) or one independent replicate (G2-to-G1 ratio) ± SEM. **P < 0.01 calculated by one-way ANOVA followed by Dunnett’s multiple comparisons test relative to the solvent control

The cell cycle phase distribution of differentiated and proliferating RPTEC/TERT1 and mProx24 after 24 h treatment at PhysOx with 0, 5 and 25 µM tolvaptan was studied by staining the cells with propidium iodide and analysing them with flow cytometry (Fig. S3). Differentiated RPTEC/TERT1 form a compact monolayer (Aschauer et al. 2013), and therefore, the great majority (> 80%) was in the quiescent G0 phase and presented with a low G2-to-G1 ratio (0.035), as expected (Brown et al. 2003; Hardwick et al. 2015). Tolvaptan treatment did not change cell cycle distribution (Fig. 5a), in agreement with the absence of toxicity in these cells (Fig. 2a and b). On the contrary, the fast proliferating mProx24 (Fig. 5c) arrested in the G0/G1 phase at 25 µM tolvaptan (increase by 20%) and concomitantly, fewer cells were found in the S and G2 phase (G2-to-G1 ratio significantly decreased). The accumulation of mProx24 in the G1 phase after exposure to tolvaptan suggests DDR activation and corresponds with the high toxicity observed (Fig. 2a and b). Proliferating RPTEC/TERT1 (Fig. 5b) also appeared with a significantly lower population in the G2 phase at 25 µM tolvaptan (G2-to-G1 ratio significantly decreased). However, in this case, the cells did not significantly accumulate in another phase, implying that the cell cycle progression was slowed down and not halted in one specific phase.

**Fig. 6:**
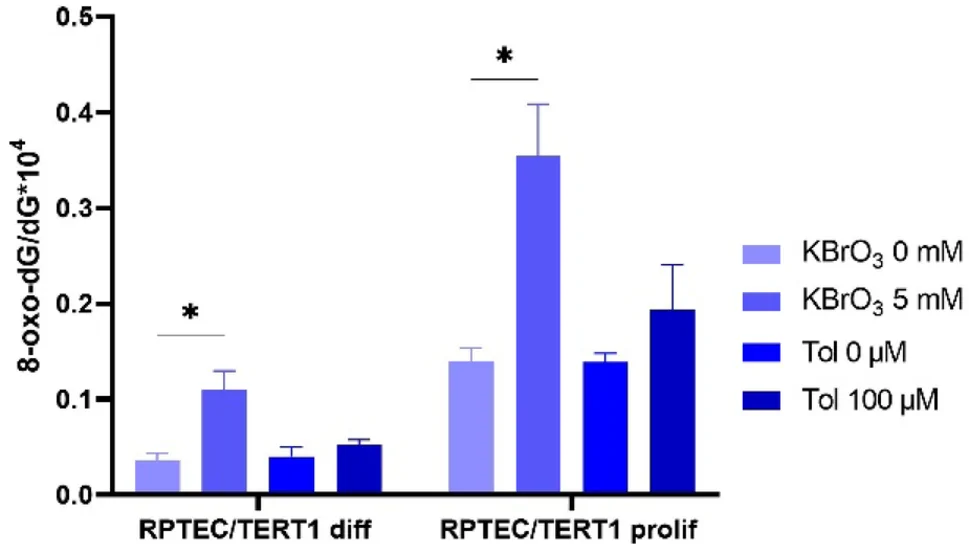
8-oxo-dG-to-dG ratio of differentiated and proliferating RPTEC/TERT1 after 90 min treatment with 100 µM tolvaptan (Tol) or after 2 h treatment with the positive control 5 mM KBrO_3_ at PhysOx as measured with LC-MS/MS. For dG measurement, the samples were diluted by a factor of 10^4^. Each point represents the mean of three independent replicates ± SEM. *P < 0.05 calculated by unpaired t-test

Next, the acute effects of ROS generation on the DNA were investigated as possible early events that lead to cell cycle arrest. The formation of 8-oxo-dG was measured with LC-MS/MS in differentiated and proliferating RPTEC/TERT1 after 90 min treatment with 100 µM tolvaptan or after 2 h treatment with 5 mM KBrO_3_ as a positive control (Kawanishi and Murata 2006; Cordero et al. 2024) at PhysOx. In differentiated RPTEC/TERT1 (Fig. 6), exposure to the positive control KBrO_3_ led to a 2.7-fold increase of 8-oxo-dG normalized to dG, while exposure to tolvaptan resulted in a 1.3-fold increase. In proliferating RPTEC/TERT1 (Fig. 6), exposure to KBrO3, similarly led to a 2.7-fold increase, while exposure to tolvaptan resulted in a 1.5-fold increase of 8-oxo-dG/dG. Although the effect of tolvaptan was not statistically significant, both proliferating and non-proliferating cells appeared to respond to ROS generation by tolvaptan with a modest increase in 8-oxo-dG levels. Albeit it should be noted that proliferating cells had a higher baseline of 8-oxo-dG/dG compared to the differentiated ones (Gorini et al. 2020), probably due to being more metabolically active and actively replicating DNA.

**Fig. 7:**
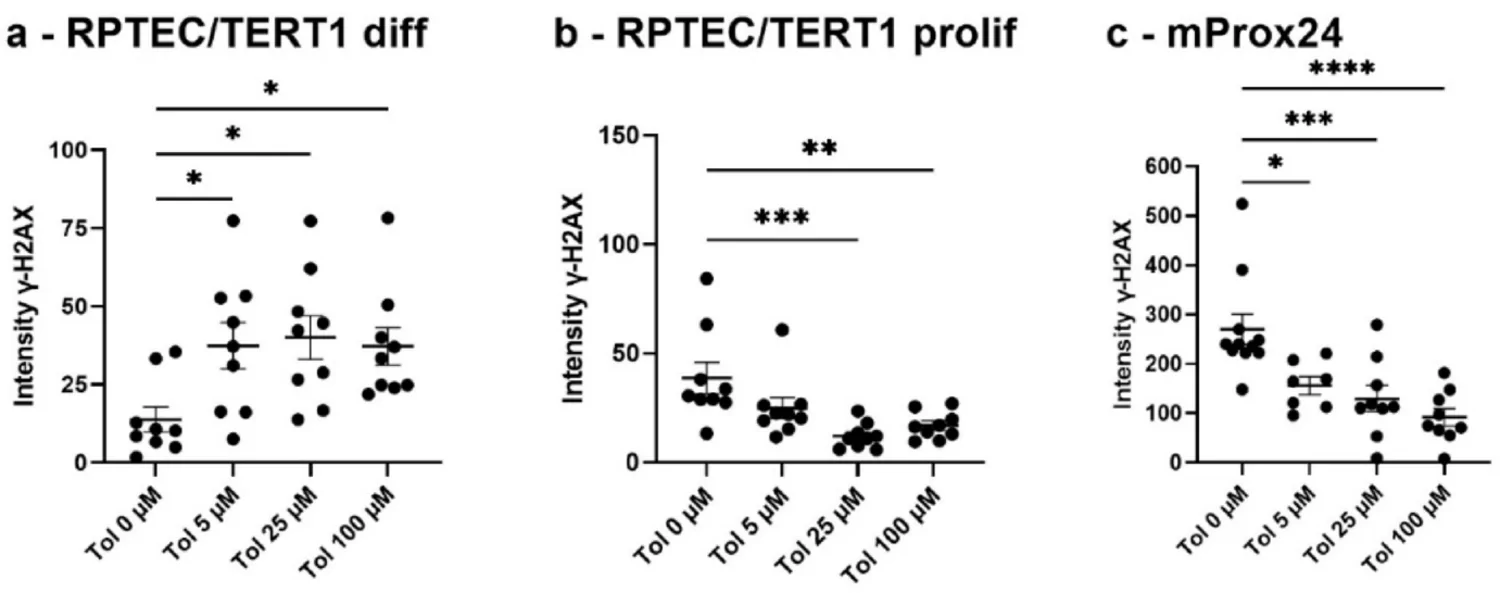
Intensity of fluorescence of γ-H2AX in the nuclei of differentiated (**a**) and proliferating (**b**) RPTEC/TERT1 and mProx24 (**c**) after 90 min treatment at PhysOx with 0, 5, 25 and 100 µM tolvaptan (Tol) as calculated from ICC images (Fig. S4, S5 and S6) with the use of ImageJ. Three independent replicates were prepared per sample and three images were captured for each replicate. Each point represents the mean intensity of one image and the lines the mean of all images ± SEM. *P < 0.05, **P < 0.01, ***P < 0.001, ****P < 0.0001 calculated by one-way ANOVA followed by Dunnett’s multiple comparisons test relative to the solvent control

Since there was a trend for higher 8-oxo-dG formation in differentiated and proliferating RPTEC/TERT1 after short treatment with 100 µM tolvaptan, γ-H2AX was selected as an early and very sensitive biomarker of DNA DSBs. In case of a DSB, the histone protein H2AX is phosphorylated and turned into γ-H2AX, as the first step of the repair process. Visualization and quantification of γ-H2AX nuclear foci are widely used to assess the DNA damage potential of compounds (Bonner et al. 2008; Mah et al. 2010). The fluorescence signal of the captured ICC images after 90 min treatment with 0, 5, 25 and 100 µM tolvaptan at PhysOx (Fig. S4, S5 and S6) was quantified and revealed that differentiated RPTEC/TERT1 increased the signal up to 3-fold compared to the control (Fig. 7a), confirming that 8-oxo-dG formation led to γ-H2AX recruitment. The saturation effect of tolvaptan, which was observed in the ATP rate (Fig. 3a), ROS/Superoxide and MMP (Fig. 4a-c) measurements, was also evident here. Interestingly, in the case of proliferating RPTEC/TERT1 and mProx24, the signal intensity of γ-H2AX was reduced after tolvaptan treatment (Fig. 7b and c). γ-H2AX is cell cycle-dependent, increasing from G1 to S/G2 phases and reaching its peak during mitosis (MacPhail et al. 2003; McManus and Hendzel 2005; Tu et al. 2013; Vallabhaneni et al. 2018). This is apparent in the present setting, as untreated differentiated RPTEC/TERT1 had a much lower baseline signal of γ-H2AX than the fast proliferating mProx24 (Fig. 7). Hence, taking into account that for example mProx24 were arrested in the G1 phase of the cell cycle (Fig. 5c) after tolvaptan treatment and after 48 h high toxicity was observed (Fig. 2a and b), a decrease in γ-H2AX levels might not necessarily reflect DNA repair, but rather changes in the cell cycle status, as suggested before (Tu et al. 2013). Notably, γ-H2AX staining of differentiated and proliferating RPTEC/TERT1 cells treated with 1000 nM bardoxolone methyl showed no difference from the solvent control (Fig. S7 and S8), further supporting the notion that the high toxicity observed (Fig. 2g and h) is not related to oxidative stress.

**Fig. 8:**
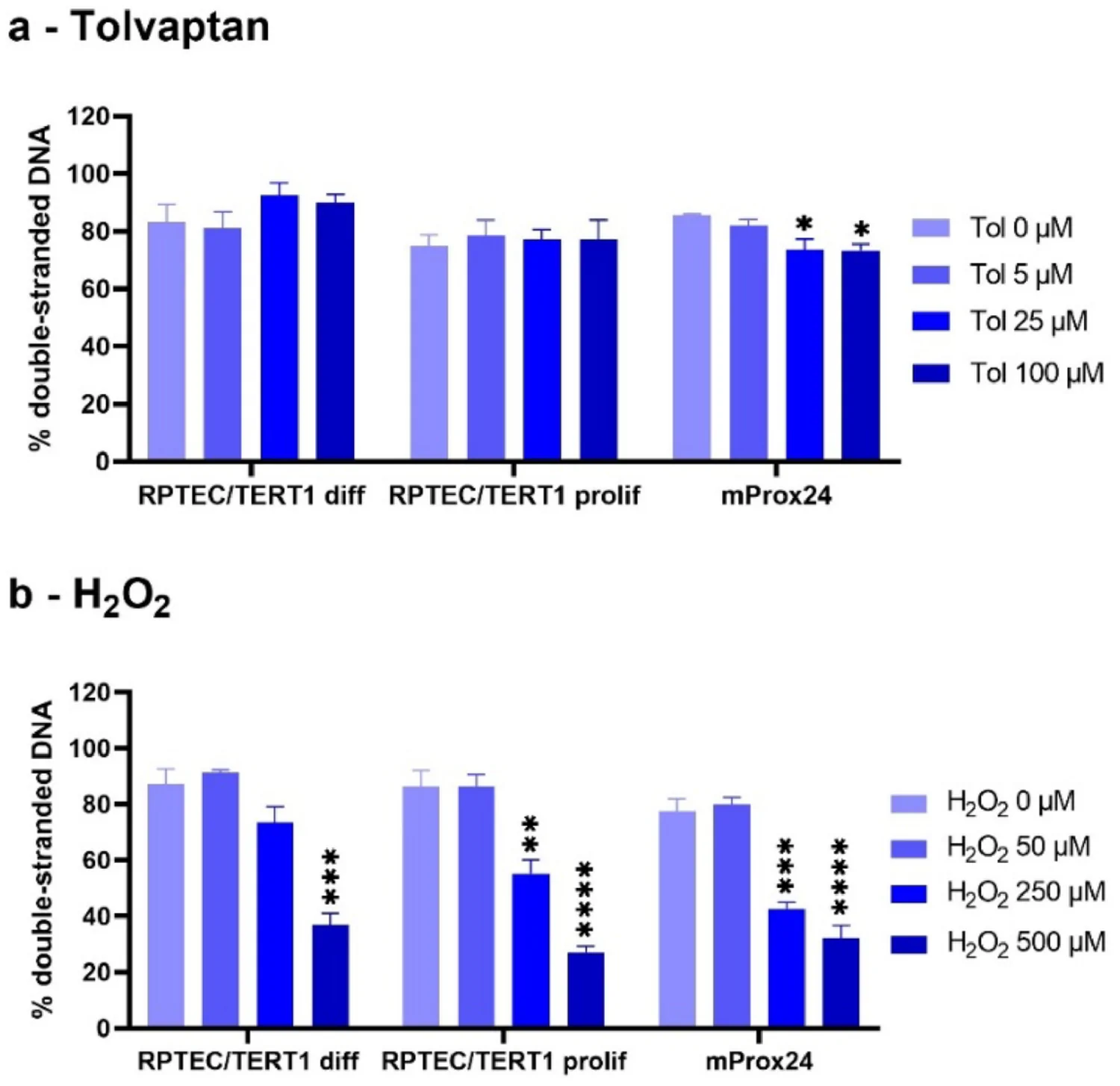
Percentage of double-stranded DNA remaining in the lysate after alkaline unwinding in differentiated and proliferating RPTEC/TERT1 and mProx24 at PhysOx after 90 min treatment with 0, 5, 25 and 100 µM tolvaptan (Tol) (**a**) or 20 min treatment with 0, 50, 250 and 500 µM H_2_O_2_ (**b**). Each point represents the mean of three independent replicates ± SEM. *P < 0.05, **P < 0.01, ***P < 0.001, ****P < 0.0001 calculated by one-way ANOVA followed by Dunnett’s multiple comparisons test relative to the solvent control

To gain deeper insight into the physical status of the DNA following exposure to tolvaptan, the potential for DNA strand break formation was studied using automated Fluorimetric Detection of Alkaline DNA Unwinding (FADU) (Moreno-Villanueva et al. 2009). The percentage of double-stranded DNA remaining after limited alkaline unwinding starting from DNA interruptions in the lysate was unchanged for differentiated and proliferating RPTEC/TERT1 after 90 min of up to 100 µM tolvaptan, and a slight but significant reduction (approximately 12%) was only observed for the mProx24 (Fig. 8a), which is also the only cell type that presented with very high cell death after 48 h tolvaptan treatment (Fig. 2b). Although the FADU assay is a tool that estimates the presence of single and double strand breaks in DNA, the differences in results observed can be attributed to the functionality and sensitivity of this method and γ-H2AX staining. γ-H2AX formation is a biological response to replication stress, stalled replication forks and DSBs that persists over several hours after recruitment to the site (Siddiqui et al. 2015; Takano et al. 2023), whereas FADU requires open-ends to initiate the limited unwinding of the DNA in the lysate under alkaline conditions and measures the state of the DNA at the certain time point of cell lysis (Moreno-Villanueva and Bürkle 2013). It is worth noting that γ-H2AX visualization has higher sensitivity as a technique (Mah et al. 2010; Reddig et al. 2018), which is seen in the present study, as H_2_O_2_ treatment induces distinct foci of γ-H2AX (Fig. S9, S10 and S11) even at concentrations that seem to have no effect with FADU (Fig. 8b). Similar to γ-H2AX staining with bardoxolone methyl, FADU also gave a negative result with all cell types (Fig. S12).

## Discussion

As most pharmaceuticals are excreted via the kidney, it is not surprising that one of the main causes of drug failure is nephrotoxicity often leading to termination of a candidate compound in development (Troth et al. 2019; Connor et al. 2024). The general term “nephrotoxicity” obscures the fact that the majority of adverse effects occur in the proximal tubule epithelial cells that express various transporters that enable the secretion and absorption processes of xenobiotics (George et al. 2017; Shen et al. 2019). More in-depth analyses of proximal toxicity demonstrated that abrogated regenerative proliferation in conjunction with epithelial toxicity forms the basis for continued progression of disease (Canaud et al. 2019; Muto et al. 2022), as for example in chronic kidney disease or interstitial fibrosis following acute or chronic insult with aristolochic acid (Yang et al. 2010).

Concomitant with the latter, it appears critical that we observed a proportional relationship between proliferation, glycolysis and toxicity in murine and human RPTEC exposed to three repurposed drug candidates for ADPKD (salicylic acid, birinapant and bardoxolone methyl) and the only available approved drug for the disease (tolvaptan) at pharmacologically relevant concentrations. Indeed, the highest sensitivity (Fig. 2) to the drugs was observed in the fast proliferating and highly glycolytic mProx24 (Fig. 1), followed by proliferating RPTEC/TERT1 and lastly by the non-proliferating and least glycolytic differentiated RPTEC/TERT1. Proliferating cells need more energy to fulfil the anabolic growth and expansion requirements, and consequently turn more glycolytic than quiescent cells (Zhu and Thompson 2019; Chandel 2021). Hence, it comes as no surprise that salicylic acid, which reduced total ATP production in a concentration-dependent manner and shifted the metabolism towards oxidative phosphorylation (Fig. 3b), followed this toxicity pattern. The pharmacokinetics and -dynamics of the compound in question can also define whether a compound will have demonstrably detrimental effects in proliferating cells. Birinapant, which directly activates apoptosis by degrading inhibitors of apoptosis proteins (IAPs) (Malas et al. 2020), was more toxic in proliferating cells than in differentiated cells, as the former would be expected to be more vulnerable to apoptosis induction during the replication process. Tolvaptan, which reduces proliferation by antagonizing vasopressin and inhibiting the ERK pathway (Reif et al. 2011; Blair and Keating 2015), also showed a similar effect. Likewise, bardoxolone methyl has been reported to inhibit nuclear factor-κB and the PI3K/Akt/mTOR pathway (Wang et al. 2017), both essential for proliferation and growth in proliferating cells (Aggarwal 2004; Park and Hong 2016; Yu and Cui 2016; Omolekan et al. 2024). Indeed, based on the previous proteomic analyses carried out (Meyer et al. 2026), RPTEC/TERT1 presented with all critical targets involved in drug-related inhibition of cell proliferation and energy homeostasis, thereby confirming the suitability of RPTEC/TERT1 for detecting drug attributed mechanism of toxicity. Similarly, cisplatin (DNA intercalating compound) and deoxynivalenol (protein synthesis inhibitor) were found to be more toxic in undifferentiated proximal tubular cells and proliferating Caco-2 cells respectively (Luo et al. 2021; Mboni-Johnston et al. 2024). Aschauer et al. ( 2015) found a significantly higher reduction of cell viability in undifferentiated RPTEC/TERT1 compared to differentiated RPTEC/TERT1 after treatment with cyclosporine A for 24 h, which was attributed to higher P-glycoprotein gene expression (higher compound efflux) in differentiated cells.

This increased susceptibility of proliferating cells was even more pronounced at PhysOx than at AtmOx (Table 1), even though the highly glycolytic PhysOx cells proliferated slower than their counterparts at AtmOx (Fig. 1). Similarly, human podocytes, multipotent mesenchymal stromal cells, cancer cells and hematopoietic stem cells have also been reported to slow down their proliferation when transferred to physiological oxygen tensions (Holzwarth et al. 2010; Eliasson et al. 2010; Hubbi et al. 2013; Hubbi and Semenza 2015; Schlichenmaier et al. 2024), as hypoxia-inducible factors (HIFs) are expected to be upregulated and induce pathways that reduce proliferation (Hubbi and Semenza 2015; Bouthelier and Aragonés 2020). It must be noted that in vivo the proliferation in the renal cortex and medulla in a healthy kidney is very low (< 1%) (Dietrich and Swenberg 1991) and increases only in case of injury for repair purposes, as evaluated by proliferation marker stainings in rat, mouse and human kidney sections (Nadasdy et al. 1994; Bonventre 2003; Oliver et al. 2009). Therefore, slower proliferation might be a reason for the higher drug sensitivity of cells at PhysOx, as they most likely cannot compensate for cell losses as fast as cells at AtmOx.

In the context of ADPKD, salicylic acid and birinapant, which have been suggested pharmacologically as repurposed drug candidates for the disease (Leonhard et al. 2019; Malas et al. 2020), reversed the cellular metabolism described for ADPKD (Warburg effect, high ATP, oxidative stress) (Podrini et al. 2020; Haumann et al. 2020), acting as an uncoupler (Fig. 4d and e) or by scavenging ROS (Fig. 4g), respectively, and thus, our data support the suitability of these drugs for the disease. Similar to our findings that salicylic acid can reverse the Warburg effect (Fig. 3b), treatment of PKD mice with salsalate (prodrug of salicylic acid) upregulated genes related to oxidative phosphorylation, the TCA cycle and fatty acid oxidation (Leonhard et al. 2019). Importantly, at the pharmacologically effective IC_50_ values (1.5 mM for salicylic acid and 50 µM for birinapant) the toxicity was low while the metabolic effects occurred at concentrations < IC_50_ values, i.e. 1 mM for salicylic acid and at 20 µM for birinapant. Especially salicylic acid provided for a large therapeutic window, as at up to 10 mM in human cells there was no or very little toxicity. However, it should be noted that only nominal concentrations were used in this study which certainly will strongly differ from the actual intracellular concentrations. Indeed, as we have shown before (Meyer et al. 2026), approximately 10% of the nominal concentration of these compounds is available intracellularly for interactions, meaning that the observed toxicity and metabolic effects in vitro occur at minimally one magnitude lower concentrations.

In contrast, bardoxolone methyl, despite slightly increasing oxidative phosphorylation (Fig. 3d), did not significantly affect parameters known to be critical for ADPKD in concentrations up to 1000 nM (Fig. 4h and i). Although bardoxolone methyl was highly toxic in proliferating RPTEC (Fig. 2g and h), our data suggest that this is not related to mitochondrial oxidative stress (Fig. 4h and i) and consequently DNA damage (Fig. S7, S8 and S12). Indeed, bardoxolone methyl (CDDO-Me) has been reported to induce apoptosis via suppression of the Akt and nuclear factor-κB pathways and mitochondrial dysfunction through loss of the MMP but not necessarily via ROS generation in contrast to its analogues CDDO and CDDO-Im that induce apoptosis in a caspase- or ROS-dependent manner (Kim et al. 2011a; Liby and Sporn 2012; Wang et al. 2017). Notably, although bardoxolone methyl had been hypothesized to ameliorate fibrosis and inflammation in ADPKD and thus had an ADPKD orphan drug designation, recently failed a phase 3 clinical trial due to lack of efficacy (Avula et al. 2022; Anand et al. 2024).

Tolvaptan, on the other hand, did reduce glycolysis (Fig. 3a), but triggered mitochondrial toxicity by hyperactivating the electron transport chain, and significantly increasing ATP, ROS/Superoxide production and the MMP (Figs. 3a and 4a-c). The hyperpolarization of the mitochondrial membrane induced by tolvaptan was reversible after co-treatment with CCCP in proliferating RPTEC/TERT1 and partially rescued its toxicity (Fig. S1), suggesting that the mitochondrial damage contributed to the observed toxic effects. The excess ROS led to DDR with distinct outcomes for differentiated and proliferating RPTEC. Differentiated cells formed 8-oxo-dG (Fig. 6) due to high ROS, and consequently, DNA damage kinases phosphorylated H2AX to γ-H2AX (Fig. 7a) to recruit repair molecules (Kinner et al. 2008; Mah et al. 2010). Taking into account that γ-H2AX does not form only in the presence of DSBs (Bonner et al. 2008; Katsube et al. 2014) and that no reduction in double-stranded DNA stemming from single- or double-strand breaks was detected by FADU (Fig. 8a), no changes in the cell cycle were present (Fig. 5a) and no cell death was observed in these cells after 48 h (Fig. 2b), we concluded that tolvaptan induced oxidative stress and a signalling response in differentiated RPTEC/TERT1, but did not lead to genomic instability and programmed cell death. In proliferating cells, ROS also led to 8-oxo-dG generation (Fig. 6), which activated DDR in the form of cell cycle arrest (Fig. 5b and c). Tolvaptan exposure has been described before in response to DNA damage to decrease in HepG2 cells at concentrations up to 100 µM the levels of cyclin D1, cyclin D3 and CDK2/4/6, which control the G1/S phase transition and cyclin B1 and CDK1 that regulate the M phase (Bertoli et al. 2013; Wu et al. 2015). Proteasomal degradation is the suggested mechanism by which tolvaptan inhibits cyclin D3 and CDK4 (Wu et al. 2015). The SV40 immortalized mProx24 cells, in which the p53-dependent responses are suppressed (Ahuja et al. 2005), crashed in the G1 phase following 25 µM tolvaptan treatment for 24 h (Fig. 5c), while they appeared with a significant increase of single- and double-strand DNA breaks (double-stranded DNA decreased) as measured by FADU after only 90 min (Fig. 8a) and massive cell death after 48 h (Fig. 2b), implying that the DNA damage was unrepairable and the cells died most likely via apoptosis (Wu et al. 2015). Proliferating RPTEC/TERT1 showed low cell death after 48 h exposure to tolvaptan (Fig. 2b), consistent with the absence of single- or double-strand DNA breaks as measured by FADU (Fig. 8a), suggesting that the cell cycle delay recorded (Fig. 5b) allowed for partial repair in a controlled fashion (Jackson and Bartek 2009). Hence, continuously proliferating cells appeared more susceptible to oxidative damage due to reduced DNA damage repair and consequently, increased genomic instability, which more likely led to higher cell death (Kaufmann and Paules 1996; Oberreuther-Moschner et al. 2005; Lee et al. 2015). This is also evident in Fig. 8b, where differentiated RPTEC/TERT1 were not as affected by H_2_O_2_ as proliferating cells.

Finally, rapamycin had no effect in any of our models, although it was tested at pharmacologically relevant concentrations (Booij et al. 2017; Malas et al. 2020).

In conclusion, we found that proliferation state, species and oxygen tension of the in vitro cell model used together with the mode of action of the compounds in question determine and drive the susceptibility of the system. The clinical significance of the differences observed between differentiated and proliferating cells is of profound importance, as this could imply that although the drugs tested would not be toxic in the healthy (differentiated) kidney, they could have a hitherto undetected nephrotoxic effect. The latter could be the case *(i)* in children and adolescents, where the kidney is still growing and thus undergoing continuous proliferation, *(ii)* when administered to an adult patient during renal injury, where they would be expected to hamper the necessary tissue regeneration and remodelling processes (Bonventre 2003), prolonging the injury phase and driving the kidney towards chronic disease (Muto et al. 2022) and *(iii)* in elderly patients, where homeostatic and regenerative cell replication is slowed down (Denic et al. 2016), thereby critically jeopardizing tissue regeneration and remodelling processes. Caution is suggested when using murine renal proximal tubule epithelial cells as surrogate in vitro systems in a NAM approach (Punt et al. 2020; Irvine et al. 2021; Schmeisser et al. 2023; Hartung and Kleinstreuer 2025) for preclinical evaluation of potentially nephrotoxic compounds, as in our case it led to an overestimation of toxicity. Culturing cells at PhysOx, even though the proliferation rate is still higher than what would be expected in vivo (Dietrich and Swenberg 1991; Nadasdy et al. 1994; Bonventre 2003), would provide a more precise estimation of the potential renal impact of drug treatment in a patient. However, more precise estimations would require more complex in vitro models (microfluidic devices, bioprinting) and introduction of the data to a physiologically relevant pharmacokinetic model of ADPKD for direct in vitro – in vivo extrapolations.

## Supplementary Information

Below is the link to the electronic supplementary material.

Supplementary Material 1

Supplementary Material 2

Supplementary Material 3

Supplementary Material 4

Supplementary Material 5

Supplementary Material 6

Supplementary Material 7

Supplementary Material 8

Supplementary Material 9

Supplementary Material 10

Supplementary Material 11

Supplementary Material 12

Supplementary Material 13

## Data Availability

No datasets were generated or analysed during the current study.
