# Supplementary material for: Species, cell proliferation state and oxygen partial pressure are major factors in the in vitro toxicity of repurposed potential ADPKD drugs in human and murine renal proximal tubule cells
Source: Arch Toxicol. 2026 Jun 27;100(11):5225–45. doi: 10.1007/s00204-026-04472-2 (PMC13694393; doi:10.1007/s00204-026-04472-2)
Supplement: Supplementary file 1 [file 204_2026_4472_MOESM12_ESM.docx]

**Supplementary Information**

**Species, cell proliferation state and oxygen partial pressure are major factors in the *in vitro* toxicity of repurposed potential ADPKD drugs in human and murine renal proximal tubule cells**

Paraskevi Sotiropoulou^1^, Andrea Gerdemann^2^, Stefan Jovanovic^3^, Hans-Ulrich Humpf^2^, Alexander Bürkle^3^ and Daniel R. Dietrich^1*^

^1^: Human & Environmental Toxicology, University of Konstanz, Konstanz, Germany

^2^: Institute of Food Chemistry, University of Münster, Münster, Germany

^3^: Molecular Toxicology, University of Konstanz, Konstanz, Germany

^*^: corresponding author: [Daniel.Dietrich@uni-konstanz.de](mailto:Daniel.Dietrich@uni-konstanz.de)

**Table 1S** LC/MS-MS parameters for 8-oxo-dG/dG determination.

| **Mass spectrometer** | QTRAP 7500 (AB Sciex) | | |
| --- | --- | --- | --- |
| **Source** | OptiFlow Pro | | |
| **Source parameter** | Ion spray voltage | | 5500 V |
|  | Source temperature | | 550 °C |
|  | Curtain gas | | 45 psi |
|  | Ion source gas 1 | | 40 psi |
|  | Ion source gas 2 | | 80 psi |
|  | Ionization mode | | positive |
|  | Scan type | | MRM |
| **Tune parameter** | Cell entrance potential | | 10 V |
|  | Cell exit potential | | 15 V |
|  | **Q1** | **Q3** | **Collision energy** |
| 8-oxo-dG | **284.1** | **168.1** | **20 V** |
|  | 284.1 | 151.0 | 50 V |
|  | 284.1 | 117.1 | 60 V |
| dG | **268.1** | **152.1** | **15 V** |
|  | 268.1 | 135.0 | 50 V |

**Table 2S** Absolute values of slopes of the cell viability and cell death curves of tolvaptan (Tol), salicylic acid (SA), birinapant (Bir), bardoxolone methyl (Brd) and rapamycin (Rap) in differentiated and proliferating RPTEC/TERT1 and mProx24 at AtmOx and PhysOx. Data is presented as mean of three independent replicates ± SEM.

| **Cell type** | **RPTEC/TERT1 differentiated** | | | | **RPTEC/TERT1 proliferating** | | | | **mProx24 proliferating** | | | |
| --- | --- | --- | --- | --- | --- | --- | --- | --- | --- | --- | --- | --- |
| **Assay** | **Cell viability** | | **Cell death** | | **Cell viability** | | **Cell death** | | **Cell viability** | | **Cell death** | |
| **Slope** | **AtmOx** | **PhysOx** | **AtmOx** | **PhysOx** | **AtmOx** | **PhysOx** | **AtmOx** | **PhysOx** | **AtmOx** | **PhysOx** | **AtmOx** | **PhysOx** |
| **Tol** | 0.128 ± 0.016 | 0.073 ± 0.032 | 0.033 ± 0.009 | 0.007 ± 0.006 | 0.173 ± 0.016 | 0.447 ± 0.058 | 0.080 ± 0.007 | 0.127 ± 0.029 | 0.981 ± 0.021 | 1.041 ± 0.010 | 0.764 ± 0.025 | 0.846 ± 0.023 |
| **SA** | 1.491 ± 0.421 | 1.347 ± 0.684 | 0.271 ± 0.095 | 0.166 ± 0.064 | 1.523 ± 0.198 | 3.474 ± 0.469 | 0.150 ± 0.075 | 0.967 ± 0.474 | 7.613 ± 0.637 | 8.748 ± 0.763 | 4.685 ± 0.415 | 5.878 ± 0.291 |
| **Bir** | 0.093 ± 0.027 | 0.061 ± 0.019 | 0.023 ± 0.010 | 0.006 ± 0.002 | 0.127 ± 0.007 | 0.200 ± 0.024 | 0.124 ± 0.010 | 0.268 ± 0.021 | 0.115 ± 0.038 | 0.341 ± 0.027 | 0.158 ± 0.027 | 0.237 ± 0.010 |
| **Brd** | 0.015 ± 0.007 | 0.006 ± 0.002 | 0.005 ± 0.000 | 0.008 ± 0.002 | 0.083 ± 0.001 | 0.089 ± 0.003 | 0.074 ± 0.002 | 0.080 ± 0.002 | 0.100 ± 0.001 | 0.112 ± 0.006 | 0.093 ± 0.001 | 0.094 ± 0.001 |
| **Rap** | 0.054 ± 0.016 | 0.069 ± 0.005 | 0.018 ± 0.007 | 0.013 ± 0.005 | 0.025 ± 0.013 | 0.066 ± 0.014 | 0.015 ± 0.013 | 0.043 ± 0.032 | 0.035 ± 0.018 | 0.062 ± 0.037 | 0.008 ± 0.001 | 0.043 ± 0.010 |

**Table 3S** Lowest Effect Concentration (LoEC) determined in reduced cell viability and increased cell death concentration-response curves of tolvaptan (Tol), salicylic acid (SA), birinapant (Bir), bardoxolone methyl (Brd) and rapamycin (Rap) in differentiated and proliferating RPTEC/TERT1 and proliferating mProx24 at AtmOx and PhysOx. Numbers in brackets denote the p-values calculated via a one-way ANOVA followed by Dunnett’s multiple comparisons test relative to the solvent control.

| **Cell type** | **RPTEC/TERT1 differentiated** | | | | **RPTEC/TERT1 proliferating** | | | | **mProx24 proliferating** | | | |
| --- | --- | --- | --- | --- | --- | --- | --- | --- | --- | --- | --- | --- |
| **Assay** | **Cell viability** | | **Cell death** | | **Cell viability** | | **Cell death** | | **Cell viability** | | **Cell death** | |
| **LoEC** | **AtmOx** | **PhysOx** | **AtmOx** | **PhysOx** | **AtmOx** | **PhysOx** | **AtmOx** | **PhysOx** | **AtmOx** | **PhysOx** | **AtmOx** | **PhysOx** |
| **Tol (μM)** | >100 | 100  (0.0302) | >100 | >100 | >100 | 50  (0.0002) | 100  (0.0017) | 50  (0.0278) | 50  (<0.0001) | 25  (<0.0001) | 25  (0.0127) | 25  (<0.0001) |
| **SA (mM)** | >10 | >10 | >10 | >10 | >10 | 10  (0.0013) | >10 | >10 | 5  (<0.0001) | 5  (<0.0001) | 5  (0.0476) | 5  (<0.0001) |
| **Bir (μM)** | >100 | >100 | >100 | >100 | >100 | 100  (0.0014) | 100  (<0.0001) | 100  (<0.0001) | 100  (0.0112) | 100  (0.0008) | 100  (0.0005) | 100  (0.0334) |
| **Brd (nM)** | >1000 | >1000 | >1000 | >1000 | 250  (0.0165) | 250  (0.0094) | 500  (<0.0001) | 500  (<0.0001) | 125  (0.0002) | 125  (0.0492) | 125  (0.0198) | 125  (0.0381) |
| **Rap (nM)** | >160 | >160 | >160 | >160 | >160 | >160 | >160 | >160 | >160 | >160 | >160 | >160 |

**Fig. S1** (**a**) Fold-change relative to the solvent control of Mitochondrial Membrane Potential (MMP) of proliferating RPTEC/TERT1 at PhysOx after 90 min treatment with 50 μM tolvaptan and 1 μM CCCP alone or combined (**b**, **c**) Cell viability of proliferating RPTEC/TERT1 at PhysOx after 90 min or 24 h treatment with 50 μM tolvaptan and 1 μM CCCP alone or combined. Each point represents the mean of three independent replicates ± SEM. *P<0.05, **P<0.01, ***P<0.001, ****P<0.0001 calculated by one-way ANOVA followed by Šídák's multiple comparisons test

**Fig. S2** Fold-change relative to the solvent control of Mitochondrial Membrane Potential (MMP) and ROS/Superoxide Production of differentiated RPTEC/TERT1 at PhysOx or AtmOx after treatment with CCCP (**a**, **b**), NAC (**c**, **d**), and pyocyanin (**e**, **f**, **g**). Each point represents the mean of three independent replicates ± SEM. *P<0.05, **P<0.01, ***P<0.001, ****P<0.0001 calculated by one-way ANOVA followed by Dunnett’s multiple comparisons test relative to the solvent control (when more than two groups were compared) or unpaired t-tests (when two groups were compared)

**Fig. S3** Representative cell cycle histograms generated with FlowJo of differentiated (**a**) and proliferating (**b**) RPTEC/TERT1 and mProx24 (**c**) after 24 h treatment at PhysOx with 0, 5 and 25 μM tolvaptan

**Fig. S4** Representative ICC images of nuclei, γ-H2AX and their merge of differentiated RPTEC/TERT1 after 90 min treatment at PhysOx with 0, 5, 25 and 100 μM tolvaptan. Scale bar = 50 μm

**Fig. S5** Representative ICC images of nuclei, γ-H2AX and their merge of proliferating RPTEC/TERT1 after 90 min treatment at PhysOx with 0, 5, 25 and 100 μM tolvaptan. Scale bar = 50 μm

**Fig. S6** Representative ICC images of nuclei, γ-H2AX and their merge of mProx24 after 90 min treatment at PhysOx with 0, 5, 25 and 100 μM tolvaptan. Scale bar = 50 μm

**Fig. S7** (**a**) Representative ICC images of nuclei, γ-H2AX and their merge of differentiated RPTEC/TERT1 after 90 min treatment at PhysOx with 0 and 1000 nM bardoxolone methyl. Scale bar = 50 μm (**b**) Intensity of fluorescence of γ-H2AX in the nuclei of differentiated RPTEC/TERT1 after 90 min treatment at PhysOx with 0 and 1000 nM bardoxolone methyl as calculated from the ICC images above with the use of ImageJ. Three independent replicates were prepared per sample and three images were captured for each replicate. Each point represents the mean intensity of one image and the lines the mean of all images ± SEM

**Fig. S8** (**a**) Representative ICC images of nuclei, γ-H2AX and their merge of proliferating RPTEC/TERT1 after 90 min treatment at PhysOx with 0 and 1000 nM bardoxolone methyl. Scale bar = 50 μm (**b**) Intensity of fluorescence of γ-H2AX in the nuclei of proliferating RPTEC/TERT1 after 90 min treatment at PhysOx with 0 and 1000 nM bardoxolone methyl as calculated from the ICC images above with the use of ImageJ. Three independent replicates were prepared per sample and three images were captured for each replicate. Each point represents the mean intensity of one image and the lines the mean of all images ± SEM

**Fig. S9** (**a**) Representative ICC images of nuclei, γ-H2AX and their merge of differentiated RPTEC/TERT1 after 20 min treatment at PhysOx with 0, 50 and 250 μM H_2_O_2_. Scale bar = 50 μm (**b**) Intensity of fluorescence of γ-H2AX in the nuclei of differentiated RPTEC/TERT1 after 20 min treatment at PhysOx with 0, 50 and 250 μM H_2_O_2_ as calculated from the ICC images above with the use of ImageJ. Three independent replicates were prepared per sample and three images were captured for each replicate. Each point represents the mean intensity of one image and the lines the mean of all images ± SEM. ****P<0.0001 calculated by one-way ANOVA followed by Dunnett’s multiple comparisons test relative to the solvent control

**Fig. S10** (**a**) Representative ICC images of nuclei, γ-H2AX and their merge of proliferating RPTEC/TERT1 after 20 min treatment at PhysOx with 0, 50 and 250 μM H_2_O_2_. Scale bar = 50 μm (**b**) Intensity of fluorescence of γ-H2AX in the nuclei of proliferating RPTEC/TERT1 after 20 min treatment at PhysOx with 0, 50 and 250 μM H_2_O_2_ as calculated from the ICC images above with the use of ImageJ. Three independent replicates were prepared per sample and three images were captured for each replicate. Each point represents the mean intensity of one image and the lines the mean of all images ± SEM. ****P<0.0001 calculated by one-way ANOVA followed by Dunnett’s multiple comparisons test relative to the solvent control

**Fig. S11** (**a**) Representative ICC images of nuclei, γ-H2AX and their merge of mProx24 after 20 min treatment at PhysOx with 0, 50 and 250 μM H_2_O_2_. Scale bar = 50 μm (**b**) Intensity of fluorescence of γ-H2AX in the nuclei of mProx24 after 20 min treatment at PhysOx with 0, 50 and 250 μM H_2_O_2_ as calculated from the ICC images above with the use of ImageJ. Three independent replicates were prepared per sample and three images were captured for each replicate. Each point represents the mean intensity of one image and the lines the mean of all images ± SEM. ****P<0.0001 calculated by one-way ANOVA followed by Dunnett’s multiple comparisons test relative to the solvent control

**Fig. S12** Percentage of double-stranded DNA in differentiated and proliferating RPTEC/TERT1 and mProx24 at PhysOx after 90 min treatment with 0, 200, 500 and 1000 nM bardoxolone methyl. Each point represents the mean of three independent replicates ± SEM
